# The Impact of Visual Elements of Packaging Design on Purchase Intention: Brand Experience as a Mediator in the Tea Bag Product Category

**DOI:** 10.3390/bs15020181

**Published:** 2025-02-09

**Authors:** Chang Liu, Mat Redhuan Samsudin, Yuwen Zou

**Affiliations:** 1 College of Creative Arts, Universiti Teknologi MARA (UiTM), Kelantan Branch, Kota Bharu 15050, Kelantan, Malaysia; 2 College of Creative Arts, Universiti Teknologi MARA (UiTM), Kelantan Branch, Machang 18500, Kelantan, Malaysia; redhuansamsudin@uitm.edu.my; 3 Faculty of Communication and Media Studies, Universiti Teknologi MARA (UiTM), Shah Alam 40450, Selangor Darul Ehsan, Malaysia; 2024373967@student.uitm.edu.my

**Keywords:** packaging design, visual elements, brand experience, purchase intention, orthogonal experiment

## Abstract

While packaging design plays a vital role in experience-oriented markets, how multiple visual elements influence purchase intention through brand experience remains unclear. This study addresses this gap by employing innovative orthogonal experiments to examine the complex relationship between the visual elements of packaging design and purchase intention for low-involvement products, integrating both design and marketing perspectives. Through orthogonal experimental design, we developed 14 packaging prototypes as stimuli by systematically manipulating five visual elements (Colour, Graphics, Logo, Typography, and Layout). The framework and prototypes were validated through expert evaluation. Data were collected via a cross-sectional survey from 490 tea bag consumers and analysed using SPSS (version 29.0) for preliminary data processing and Mplus (version 8.3) for structural equation modelling. Our results reveal the direct effects of visual packaging elements on consumer purchase intention. Notably, Colour, Graphics, Logo, and Layout significantly influence purchase intention through brand experience mediation. Importantly, our multi-level analysis of visual elements unveils distinct patterns in how different design levels (e.g., colour harmony, graphic types) affect consumer responses. This study provides novel theoretical insights into how consumers make purchase decisions based on packaging design visual elements, addressing a significant gap in existing research. Unlike previous studies focusing on isolated design elements, our systematic classification and multi-level analysis offer both theoretical insights into packaging design mechanisms and practical guidelines for designers and practitioners.

## 1. Introduction

In the global consumer goods market, packaging design has evolved from a simple product protection function to a key strategic tool for brand competition. This evolution is particularly evident in China’s fast-growing packaging market, which, according to the *China Packaging Market Size, Analysing Material Type, Innovations and Forecast to 2028* report published by GlobalData (2024), reached 912.9 billion units in 2023 and is expected to grow at a compound annual growth rate of more than one percent from 2023 to 2028. The strong growth in the output value of China’s packaging industry not only reflects high demand but also fierce competition in the current market. A global consumer survey conducted by Ipsos revealed that 72 percent of consumers believe that product packaging design plays a key role in their purchasing decision-making process (Gil, 2018). Packaging has long been used as an important contact point for communication between companies and consumers throughout the life of the product, injecting vitality into the brand.

As the only brand medium that consumers experience fully 100 percent of the time, packaging delivers a higher return on investment than any other branding strategy (Wheeler, 2021). Packaging design, as an important tool for branding, has a significant impact on consumer purchase intention (PI). Studies have shown that 73 to 85 percent of consumer purchase decisions are made at the point of sale, and packaging design is often the main differentiator between products (Klimchuk & Krasovec, 2021). This importance is particularly evident in the increasingly competitive Chinese market, where packaging design has become an important part of brand strategy.

Understanding how packaging design elements influence consumer behaviour is becoming increasingly important for companies seeking a competitive advantage. Many scholars have explored the impact of different attributes of package design on PI (e.g., Benachenhou et al., 2018; Ck et al., 2022; Cortina-Mercado, 2017; Kiygi Calli & Kilic, 2020; Setiowati & Liem, 2018; Silayoi & Speece, 2007; Suci et al., 2022; Theben et al., 2020; F. Wang et al., 2022; Wulansari, 2019). It is evident that the importance of packaging as a powerful tool in brand marketing and its influence on consumer purchase decisions has been widely recognised by academics, but there are still some important research gaps that have not yet been addressed. These research gaps not only limit the theoretical understanding but also the practical application of packaging design strategies.

Firstly, current research still suffers from methodological limitations. Many studies rely primarily on questionnaires and lack the involvement of stimulus materials (Calderon-Monge et al., 2024; Zafar et al., 2022). Although this type of research has validated consumers’ general tendencies towards the concept of design elements through quantitative research methods, the limitation of most of these studies is the use of a single questionnaire without stimulus materials, which makes it difficult to accurately assess consumers’ responses to packaging design elements. More importantly, most studies on the visual design of product packaging have dealt with individual visual elements at the expense of their combined effects (Martinez et al., 2021; Pleyers, 2024; Spence & Van Doorn, 2022). Consequently, most current studies have somewhat limited practical applicability in packaging design strategies and cannot provide appropriate and specific guidance for packaging design strategies.

Secondly, existing research has specific limitations in testing the impact of visual elements of packaging design (VEPD) at different levels. Research has generally explored the impact of specific colours such as red or blue on consumer behaviour (Casales-Garcia et al., 2024; J. Su & Wang, 2024; Van Esch et al., 2019), yet research on the application of systemic colour systems remains limited. Similarly, research on graphics elements of packaging is contradictory, with Thomas and Capelli (2023) demonstrating that repeated figurative graphics can significantly influence both the intended and actual product experience, thereby affecting PI, while some scholars maintain opposing views. For logo elements of packaging, while some studies have provided insights into how logo design features influence consumer behaviour (Henderson & Cote, 1998; Henderson et al., 2003; Haberstroh et al., 2018), these studies have generally failed to synthesise the individual and combined effects that could optimise consumers’ overall brand perception and PI. Regarding the typography element of packaging, there is a particular need to study Chinese fonts in the local context, given that font preferences and perceptions vary across countries and regions (Huang et al., 2023). Although recent research has emphasised the importance of layout in product usability (Zhao et al., 2024), systematic evaluations of the impact of different levels of layout design on PI remain scarce.

Furthermore, while there has been extensive research on the impact of packaging on consumer PI, little is known about how consumers generate PI through brand experience (BE). Among existing studies, researchers have focused on the impact of brand touchpoints on BE and customer decision-making regarding intangible touchpoints such as products and services, while research on tangible touchpoints such as packaging is particularly limited (Silva et al., 2020; Stein & Ramaseshan, 2016). Notably, this gap is particularly evident in the Chinese research context, where the relationship between VEPD and BE remains largely unexplored. Specifically, the mechanisms of how packaging design influences consumer decision-making through BE remain under-explored (Antunes & Veríssimo, 2024; Rehman & Elahi, 2024).

To sum up, the research on the influence of VEPD on consumers’ purchasing decisions is concentrated in the field of marketing, and there is still no consensus on the division of VEPD in academic circles. The discussion on VEPD remains one-sided. From another perspective, in the field of design, most studies on the influence of VEPD on purchasing decisions are based on empirical qualitative analyses, and accordingly, their objectivity is limited to a certain extent. Although the importance of packaging as a powerful weapon of brand marketing and its influence on consumers’ purchasing decisions has been widely recognised by academics, few studies have been conducted on the impact of VEPD on consumers’ purchasing decisions. Moreover, there are few studies that integrate design and marketing to focus on the tangible VEPD in brand touchpoints from a multidisciplinary perspective and study the complex relationship and specific mechanism between VEPD and consumers’ PI.

The study of the above content can not only provide designers with corresponding design strategies and scientific basis when designing packaging but can also provide a feasible path for the use of experiential marketing in packaging design. Therefore, it is necessary to conduct this study to verify the relationship between the above variables. This will provide designers and marketers with more comprehensive insights aimed at optimising packaging strategies in line with changing consumer preferences. Therefore, this study bridges the current research gap by pursuing the following research objectives:

RO1: To systematically investigate the combined effects of visual elements of packaging design through an orthogonal experimental design.

RO2: To investigate the mediating role of brand experience in the process of influencing consumers’ purchase intention by visual elements of packaging design.

This study is structured as follows: Section 2 presents the research in the field to date through a literature review, followed by the research hypotheses. Section 3 presents the stimulus materials and measurement tools used in this paper and describes the application of the methodology. Section 4 presents the results of this study. The results are discussed in Section 5. Finally, in Section 6, the theoretical and practical implications are described, and the limitations of the study and directions for future research are highlighted.

## 2. Literature Review and Hypothesis Development

### 2.1. Theoretical Framework

This section reviews and discusses the key underpinning theories involved in this study, namely the stimulus–organism–response (S-O-R) theory, the visual design elements theory, and the brand experience model theory. These theories form the basis for the development of the conceptual framework of this study.

#### 2.1.1. Stimulus–Organism–Response Theory

The S-O-R theory, as an important theory in psychology and behavioural sciences, has been widely used in the field of design and marketing research (Köseoğlu & Tuncer, 2023). The theory usually focuses on areas such as online or offline shop environment design, website visual design, and mobile application platform design attributes. However, there is still a lack of research in the area of packaging design elements and consumer behaviour, which is where the current research gap in the field lies.

Existing studies do not focus on investigating different product categories and consumer segments and do not provide a detailed exploration of specific packaging design elements. For example, Briand Decré and Cloonan (2019) investigated how package glossiness affects consumers’ tactile perception and behavioural intentions, and did not address the impact of other package design elements, such as colour, graphics and typography, etc. C. Pan et al. (2021) investigated how green packaging indirectly affects green PI through the mediation of perceived value and green satisfaction, without considering other design elements and focusing only on environmental attributes. Housni et al. (2023) analysed the determinants of intention for terroir products. The study emphasised the influence of terroir of origin, terroir labelling, and technology as environmental cues on consumer perceptions of product labelling on PI, but ignored the combined effect of various packaging design elements.

Given the limitations of the above studies and the lack of exploration of not only different packaging design elements but also the impact of specific packaging design elements on different types of products and consumer segments, these research gaps suggest that there is still a wider scope of research in the field for researchers to explore, especially in such a competitive consumer market. A more in-depth exploration of packaging design elements and how they comprehensively influence consumer decision-making based on the S-O-R theory would be of great academic value and practical significance.

Therefore, the S-O-R theory serves as a practical and fundamental framework to construct a conceptual framework to validate the research questions of this study, which not only refines the specific mechanism of the influence of packaging design elements on consumer behaviour but also further extends the application scope of this theoretical framework. This study can provide a deeper understanding of how packaging influences consumers’ purchasing decisions and help companies to optimise their packaging strategies for better market performance. Based on this theory, the conceptual framework of this study will be composed of three main parts: stimulus, organism, and response. Specifically, the VEPD are an external stimulus, which affects the consumers’ internal mediating constructs (i.e., BE), and then influences their behavioural response (i.e., PI).

#### 2.1.2. Visual Design Elements Theory

Visual design elements theory can be traced back to the rise of the modernist movement in the early 1900s. At the end of the 1920s, the modernist movement reached its climax and developed in full swing in Germany, the Netherlands, the Soviet Union, and several other European countries. Especially in Germany, extremely important results were achieved through the explorations of the Bauhaus school (Zhang, 2009).

The composition of visual design is an important part of visual expression, which can be better expressed by clarifying the composition of visual elements. As for the classification of visual elements, there are two classifications based on different perspectives: one is the classification of traditional design elements, i.e., line, shape, colour, texture, space, and typography (Wong, 1993); and the other is the classification of modern design elements, i.e., colour, graphics, logo, typography, and layout. The former emphasises the basic composition of visual design, while the latter emphasises the application of visual design. For example, line, shape, space, and other elements can constitute graphics, layout, and other elements. In other words, the former provides a theoretical basis for visual design, and the latter, as an important component of modern visual design, forms the basis of graphic design through the combination and application of these elements, used to create visually compelling and functionally effective design works, which focuses on the specific application of design elements in design practice and their effects.

At the current intersection of design and marketing, modern design elements (visual design elements such as colour, graphics, and typography) are becoming increasingly important, not only because they are an essential part of design theory but also because they have demonstrated their applicability in many studies involving applied research and consumers (Celhay et al., 2017; Hazwani et al., 2021; Murchie & Diomede, 2020; Ozretić Došen & Brkljačić, 2018; Z. Pan et al., 2024). Therefore, based on the purpose of this research and visual design elements theory, this study adopts the modern classification of visual design elements that are widely recognised and applied in the current academic world, i.e., colour, graphics, logo, typography, and layout.

Although VEPD is widely recognised as a key application area for visual design elements theory, there are still limitations in research in this area. A review of the existing literature reveals that most scholars have focused on qualitative analyses of the VEPD. For example, some researchers investigated the classification and principles of graphic design elements in packaging. Spence and Van Doorn (2022) reviewed the importance of colours and visual imagery in the design of food and beverage packaging. However, few scholars have focused on the VEPD at a validation level to fully validate the psychometric properties of having the aforementioned elements. Compared to these qualitative approaches, quantitative research on the psychometric properties of visual elements is still limited. For example, E. S.-T. Wang (2013) investigated the impact of visual package design on consumer perceptions, showing that the attitude of visual packaging influences consumers’ perceptions of food quality and brand preference. Sook et al. (2020) empirically verified the significant effect of visual elements of packaging on PI. These studies emphasise the need for more systematic quantitative research methods when studying packaging design elements.

Although the above studies validate the psychometric properties of the overall concept of VEPD, they have neglected the specific manifestations of different visual design elements and lacked detailed analysis and research on specific sub-elements. In particular, the specific roles and influence mechanisms of individual visual elements (e.g., colour, graphics, typography) at different levels and design forms need to be explored to fully understand their contribution to brand perception and consumer behaviour. In other words, it is important to explore the specific characteristics of VEPD, the mechanisms of influence through which they occur, and the conditions under which each characteristic does or does not influence consumer behaviour, as this will contribute to a more accurate understanding of the specific impact of visual design elements. This will not only help to improve the accuracy of design theories but also provide more scientifically based guidance for design practice. Therefore, based on visual design elements theory, this study will construct a theoretical model of the VEPD (colour, graphics, logo, typography, and layout) and consumer behaviour with a view to fully validating the relationship between the aforementioned elements at the validation level.

#### 2.1.3. Brand Experience Model Theory

The development of brand experience model theory can be traced back to research in marketing and consumer behaviour, which emphasises the multidimensional experience of brand–consumer interaction. The concept of BE was first introduced by Schmitt (1999), emphasising the importance of BE in shaping consumer perceptions and behaviours, and has been further developed and refined in subsequent research. This theory delves into the interaction between brands and consumers based on how consumers experience brands through multiple dimensions of sensory experience (SE), affective experience (AE), intellectual experience (IE), behaviour, and relationships (Schmitt, 1999). With the intensification of market competition and the diversification of consumer needs, the concept of BE began to receive gradual attention in the 1990s.

Previous research on brand touchpoints and BE has focused on intangible touchpoints. For example, Venter de Villiers et al. (2018) examined the impact of a shop environment on PI through BE in fashion retailing. However, research on packaging as a tangible brand touchpoint is still limited. A study by Variawa (2010) explored packaging and BE as purchase decision criteria for low-income consumers of fast-moving consumer goods, and found that packaging affects BE differently across price segments. However, comprehensive studies examining the relationship between packaging design elements and BE, particularly in a broader consumer context, remain scarce. This gap is particularly evident given the role of packaging as a key physical touchpoint in brand–consumer interactions.

Brand touchpoints serve as key antecedent variables of BE, and understanding which factors are most effective in triggering a positive BE can help companies and brands to make more targeted strategies when designing touchpoints. However, packaging, as one of the key touchpoints of a brand, is usually the first point of contact for consumers and plays a key role in consumer decision-making. However, there is a lack of research in the literature on the impact of packaging design elements on BE, especially VEPD. Therefore, it is necessary to explore how the VEPD affects BE and further enriches the application of brand experience theory in the field of packaging design. It is worth noting that this study focuses on the VEPD, and therefore the stimulus categories involved in the study focus on sensory, affective, intellectual stimuli, and behavioural stimuli are not discussed.

### 2.2. Hypothesis Development

**Figure 1:**
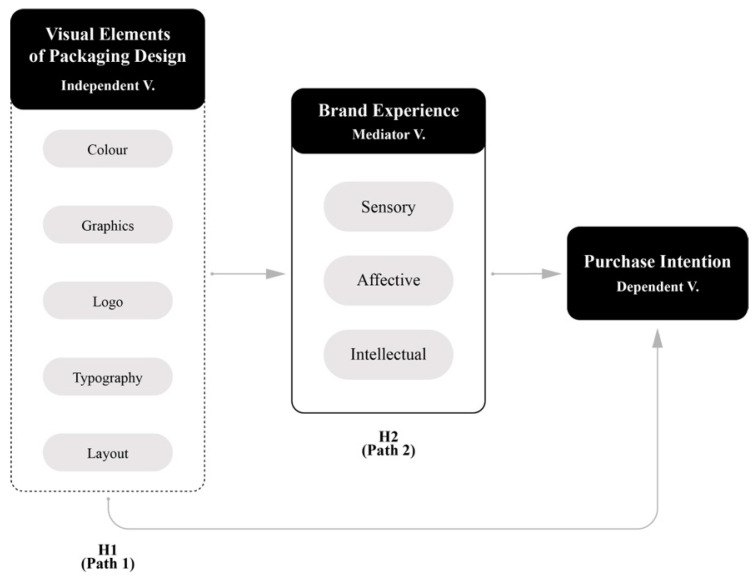
Research conceptual framework. Source: author’s drawing.

The following subsection describes the hypotheses of the conceptual framework, which is illustrated in the conceptual framework diagram shown in Figure 1, and which covers the VEPD, BE, and PI. Previous empirical findings on the relationships between the variables are presented to support the hypotheses and the relationships between the variables are discussed in detail below.

#### 2.2.1. The Relationship Between Visual Elements of Packaging Design and Purchase Intention

According to the theory review above, visual design elements are an important part of visual expression, and by clarifying the composition of visual elements, it is possible to make a better visual expression and understand the actual impact of each visual element of packaging design on consumers’ attitudes. Based on the visual design elements theory and through integrating the discussions of related researchers on the elements of packaging design, this study divides the VEPD into five elements: colour, graphics, logo, typography, and layout.

Existing studies have specific limitations in testing the impact of visual packaging design elements at different levels. Specifically, with regard to colour, although there are existing studies covering the influence of colour on consumer behaviour, these studies tend to ignore the importance of the colour system theory in its practical application. The studies by Pereira et al. (2022) and Yu et al. (2023) attempted to influence consumers’ purchasing decisions through colour expectations and associations, and Bezaz and Kacha (2021) attempted to influence consumers’ packaging evaluations and brand attitudes through the three elements of colour (hue, brightness, and saturation). However, none of them have fully utilised a colour system such as the Practical Colour Coordinate System (PCCS) to systematically assess and optimise these effects. Therefore, investigating the effects of VEPD on PI under the PCCS colour system can not only fill the gap of existing research, but can also provide designers and marketers with more scientific and operational design strategies (Casales-Garcia et al., 2024; Felix et al., 2022). As the PCCS colour system combines the advantages of the Munsell system and the Ostwald system, it combines the brightness and purity of colour as a hue, and forms a corresponding colour impression system, which makes colour matching intuitive, fast, and easy to operate. At the same time, the PCCS colour system is also more suitable for the practical application and matching of colour. Therefore, this study will be based on the PCCS colour system to divide the visual element of colour into levels. According to the PCCS harmony rule, the colour element can be divided into three levels, namely monochromatic harmony, analogous harmony, and contrast harmony.

With respect to graphics, while the current literature provides a solid foundation for understanding the impact of packaging graphics elements, it often neglects comparative analyses of graphics types (figurative, abstract, and hybrid) within a consistent experimental framework. This oversight highlights a critical research gap: a comprehensive study is needed to assess the relative effectiveness of these graphic types in influencing consumer purchase intent. At the same time, the use of graphics in packaging design is not limited to figurative graphics or abstract graphics; the use of figurative graphics and abstract graphics are not opposites. Designers can carry out the juxtaposition and combination of multiple visual elements according to the purpose and needs of the design, including the hybrid use of figurative and abstract aspects (He, 2018). However, it is worth mentioning that hybrid graphics, as one of the important categorisations of graphics design elements, are often overlooked by scholars. Therefore, addressing this research gap is not only of academic interest, but also provides practical implications for marketers and designers seeking to optimise packaging strategies to better meet consumer expectations and improve market performance (Fenko et al., 2018; Maleki et al., 2020).

For logo elements, while the strategic importance of logo elements in packaging design is widely supported by relevant research, there is still a gap in the overall understanding of how different logo types specifically influence consumer behaviour. This highlights an important oversight in current research—the need for a more nuanced examination of how different logo types influence a wider group of consumers. In-depth research into the strategic use of logomark, logotype, and combination mark in specific market segments can therefore provide designers and marketers with specific design guidelines and marketing strategies to scientifically optimise logo elements and enhance consumer purchase motivation.

Regarding typography, although typography has a potential that should not be ignored in promoting consumer PI, how to select the appropriate type of typography according to specific market needs remains a question that needs to be further explored. This study aims to fill this research gap by systematically evaluating consumers’ responses to different Chinese character typography arrangements to clarify the exact role of typography choice in the consumer purchase process. This will not only enrich existing visual merchandising theories, but also provide guidance for packaging design diversification in practice, especially in highly culturally specific market environments such as China (Boon & Bozinovski, 2019; Grohmann et al., 2013; Haberstroh et al., 2018; Liu et al., 2019). Given the lack of research on the mechanism of the influence of typography on consumer behaviour in packaging design in the Chinese context, this study will fill this research gap by investigating the influence of typography on consumer behaviour in the Chinese context based on Chinese character print typeface, calligraphy typeface, and artistic typeface.

Regarding layout, while existing research provides a preliminary understanding of layout design, there is insufficient research on the unique impacts of different layouts, which may be fundamentally different in terms of visual presentation and efficiency of information transfer, and there is a need to distinguish between the unique impacts of different layout designs (Otterbring et al., 2013; Silayoi & Speece, 2007). A grid layout system is a tool used to organise content in visual design by arranging different graphic design elements by dividing the page into units or zones (Robins et al., 2010). Scholars have given different perspectives on the division of structural systems or known as choreographic forms. Kang (2014) summarised the forms of choreography into eleven types: standard, symmetrical, left-right placement of pictures, repetitive, free-form, centre-axis, four-point, textual, top-bottom-spanning, word-illustration, and instructional. Because its classification guidelines are confusing and unsystematic, its division is slightly lacking. Elam (2018) described eight types of layout systems, which are an axial system, radial system, dilatational system, random system, grid system, transitional system, modular system, and bilateral system. Although the classification criteria are clear and systematic, some of these layout systems are not applicable to packaging design, so this study cannot copy the classification in its entirety. Therefore, this study will be based on Elam’s (2018) division, and combined with packaging design practice, the layout elements will be divided into four levels, which are bilateral symmetry, grid, centralised, and diagonal.

In summary, due to the lack of research on the effects of different levels of VEPD on consumers’ PI as mentioned above, this study will rely on previous relevant studies and the results on consumers’ preference for visual design elements and propose hypotheses based on the relevant studies mentioned above.

**H1.**  *The visual elements of packaging design have a significant direct influence on consumers’ purchase intention.*

**H1a.**  *The colour elements of packaging design have a significant direct influence on consumers’ purchase intention.*

**H1b.**  *The graphics elements of packaging design have a significant direct influence on consumers’ purchase intention.*

**H1c.**  *The logo elements of packaging design have a significant direct influence on consumers’ purchase intention.*

**H1d.**  *The typography elements of packaging design have a significant direct influence on consumers’ purchase intention.*

**H1e.**  *The layout elements of packaging design have a significant direct influence on consumers’ purchase intention.*

#### 2.2.2. The Mediating Role of Brand Experience in the Relationship Between the Visual Elements of Packaging Design and Purchase Intention

In recent years, scholars have gradually recognised that BE not only directly affects consumers’ purchasing decisions, but may also act as a mediating variable that influences the effectiveness of other marketing activities (Gao & Shen, 2024; Khan & Rahman, 2015; Moreira et al., 2017; Rather et al., 2024; Venter de Villiers et al., 2018; Yang & Chen, 2010). In addition, visual attributes have the most significant impact on BE among all senses, especially in explicit visual perception, which has high relevance for both SE and IE, thus visual attributes play a key role in BE and brand-related performance indicators (Haase et al., 2018). In the field of packaging design in particular, studies have shown that visual elements of packaging can significantly enhance BE and thus influence consumers’ PI (Nivedhitha & Manzoor, 2020; Shukla et al., 2022).

However, while the importance of BE in shaping consumer behaviour and enhancing brand loyalty is widely recognised, there has been some progress in the current literature in exploring the ’impact of VEPD on BE’ (Gao & Shen, 2024; Nivedhitha & Manzoor, 2020; Shukla et al., 2022; Variawa, 2010). For example, Variawa (2010) examines the impact of packaging aesthetics on BE but does not delve deeper into how this experience translates into purchase behaviour. Similarly, Gao and Shen (2024) identify the impact of SE on brand loyalty, but do not adequately analyse how this experience improves consumers’ purchasing decisions through packaging design. Thus, the mediating role of BE between VEPD and PI remains under-researched.

Therefore, the mediating role of BE in the pathway of ‘VEPD–BE–PI’ has not yet received sufficient empirical support. Although the existing literature provides a theoretical basis for the multifaceted effects of BE (Amer et al., 2023; Chen et al., 2021; Schmitt, 1999), there is still a research gap to be filled in terms of the specific mechanisms by which VEPD influences consumers’ PI through BE. This research gap suggests the importance of further exploring how BE acts as a bridge between packaging design and consumer PI. Especially in the rapidly developing consumer market, understanding this mediating mechanism can provide designers and marketers with key strategic guidance to create more effective consumer touchpoints to improve market competitiveness and consumer satisfaction, and can provide new paths for the in-depth development of marketing theories in practical applications.

Due to the lack of a clear framework of the mediating role of BE in the relationship between VEPD and PI, this study will rely on previous studies and results related to the mechanism of the role of BE as a mediating variable and propose the hypothesis based on the relevant studies mentioned above.

**H2.**  *Consumers’ brand experience plays a significant mediating role in the relationship between the visual elements of packaging design and purchase intention.*

**H2a.**  *Consumers’ brand experience plays a significant mediating role in the relationship between the colour elements of packaging design and purchase intention.*

**H2b.**  *Consumers’ brand experience plays a significant mediating role in the relationship between the graphics elements of packaging design and purchase intention.*

**H2c.**  *Consumers’ brand experience plays a significant mediating role in the relationship between the logo elements of packaging design and purchase intention.*

**H2d.**  *Consumers’ brand experience plays a significant mediating role in the relationship between the typography elements of packaging design and purchase intention.*

**H2e.**  *Consumers’ brand experience plays a significant mediating role in the relationship between the layout elements of packaging design and purchase intention.*

## 3. Materials and Methods

### 3.1. Stimuli

To obtain suitable stimulus materials (i.e., packaging design prototypes) for validating research hypotheses, this study classified VEPD into five factors. Based on the previous literature, these factors include colour with three levels (monochromatic harmony, analogous harmony, contrast harmony) (Asakura, 2018; Lupton & Phillips, 2009), graphics with three levels (figurative, abstract, hybrid) (He, 2018; Klimchuk & Krasovec, 2021) logo with three levels (logomark, logotype, combination mark) (Nöth, 1997; Wheeler, 2021), typography with three levels (Chinese character print typeface, calligraphy typeface, artistic typeface) (Kan, 2012; K. Su, 2015), and layout with four levels (bilateral symmetry, grid, centralised, diagonal) (Elam, 2018; Poulin, 2011). The detailed categorisation of each factor and level is presented in Table 1.

If the five factors and their corresponding levels underwent a full-scale test, the number of packaging samples required would be 3 × 3 × 3 × 3 × 4 = 324. Conducting a full-scale test in this study would be impractical and inefficient (Fang et al., 2018). However, the orthogonal test method, based on mathematical principles and orthogonality, selects factors and levels with orthogonal properties from numerous test factors, significantly reducing the number of tests while maintaining effectiveness. It is a scientific and efficient method for researching and handling multifactorial tests (Fraley et al., 2024). Therefore, this study employed the orthogonal test method for developing packaging design prototypes, balancing factors and levels while reducing the number of trials, thus ensuring the representativeness of the packaging design prototypes.

This study follows a systematic orthogonal experimental design process for developing packaging design prototypes. Firstly, through the literature review, we identified the VEPD factors and their levels. Secondly, we selected and designed an orthogonal table adapted for this study. While traditional orthogonal tables require consistent levels across factors, our five factors had varying numbers of levels. Therefore, to accommodate this mixed orthogonal design, we adapted the standard orthogonal table using the proposed level method and combination method, designing a suitable mixed orthogonal table. Thirdly, we mapped the hybrid orthogonal table to factors and levels, checking for missing factor level combinations to obtain the final hybrid orthogonal table, as shown in Table 2.

Following the VEPD framework and hybrid orthogonal tables, we created 14 packaging design prototypes using Adobe Illustrator (version 26.4.1) and Adobe Photoshop (version 24.0.0). Each prototype systematically varied in visual elements while maintaining professional quality and consistency. The prototypes were presented as high-resolution images (6000 × 4500 pixels) on a neutral background, ensuring high-quality visual presentation and eliminating distractions for participants focusing on the stimuli.

### 3.2. Expert Validation

To ensure research design validity, we conducted expert validation with six experts, comprising senior academics and designers in packaging design and consumer behaviour research. The experts evaluated our VEPD framework, stimuli design, and measurement instruments using a structured assessment tool (five-point scale, 1 = strongly disagree, 5 = strongly agree).

**Figure 2:**
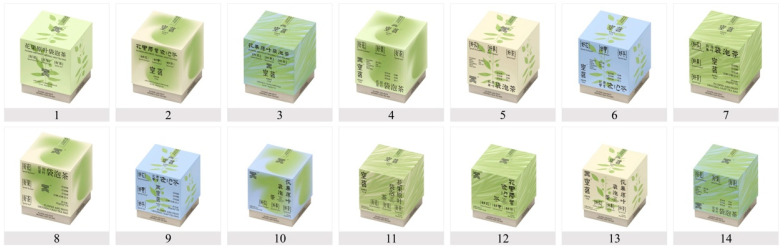
Stimulus materials used in the study. Items 1–14 represent packaging prototypes, comprising the brand name (Kong Tea), product category (Flower and Fruit Original Leaf Tea Bag), and brand slogan (Good Flowers, Good Fruits, Good Tea). Source: author’s drawing.

Expert validation supported the research design. The VEPD framework’s element classification system received high ratings (M = 4.56, SD = 0.31), with strong validation for individual elements: colour (M = 4.78, SD = 0.25), graphics (M = 4.50, SD = 0.32), logo (M = 4.67, SD = 0.38), typography (M = 4.28, SD = 0.36), and layout (M = 4.17, SD = 0.42). The framework’s practical applicability was confirmed (M = 4.72, SD = 0.30), indicating theoretical soundness and practical value. Expert evaluations of the 14 packaging design prototypes showed high inter-rater agreement, with mean ratings from 4.83 to 4.94 (SD ranging from 0.09 to 0.19), suggesting strong consensus on the appropriateness and validity of all design manipulations (stimuli shown in Figure 2). Based on expert feedback, we enhanced the measurement instrument by including shopping scenario descriptions to facilitate respondents’ perception expression.

### 3.3. Variable Operationalisation and Measurement

To investigate VEPD effects, key variables were manipulated and measured as follows. Given the categorical nature (multifactorial and multi-level nominal variables) of VEPD (independent variables), this study employed effect coding to transform qualitative data into quantitative indicators suitable for structural equation modelling (SEM) analysis (Sundström, 2010). This coding scheme enables a comparison of each category to the overall average (Alkharusi, 2012; Cohen et al., 2015; Graves & Merkle, 2021; MacGregor et al., 2022; Sin, 2011) while ensuring design orthogonality and the independent estimation of different factors’ effects (Montgomery, 2017).

In the effect coding system, similar to dummy coding, n-1 features represent n data categories. However, while the reference group is coded as 0 in dummy coding, it is coded as −1 in effect coding (Alkharusi, 2012; Cohen et al., 2015; Li et al., 2023). For visual elements with three levels, we used two coding variables where the third level was coded as (−1, −1) as the reference category (see Table A1 for the corresponding full effect coding).

BE (the mediating variable) was measured using a seven-point Likert scale, ranging from 1 (‘strongly disagree’) to 7 (‘strongly agree’). Three sub-dimensions were measured: SE, adapted from Brakus et al. (2009), Han et al. (2019), and Hwang et al. (2021); AE, adapted from Brakus et al. (2009), Ong et al. (2018), and Jeon and Yoo (2021); and IE, adapted from Brakus et al. (2009), Han et al. (2019), and Tasci and Milman (2019).

Willingness to buy (dependent variable) was measured using a seven-point Likert scale, with items adapted from Waheed et al. (2018). The wording of all scale items was modified slightly to suit this study’s context. Our pilot study confirmed satisfactory psychometric properties for these improved measures, which were subsequently adopted in the formal study (see Table A2 for measurement items).

### 3.4. Data Collection

This study utilised a non-probability sampling method and was distributed through a self-administered web-based electronic questionnaire. Reynolds et al. (2003) suggest that when targeting theoretical international research, the use of non-probability sampling methods can ensure that data obtained in different countries or cultural contexts are comparable in terms of key variables, particularly by selecting homogeneous samples that are highly similar in terms of key socio-demographic characteristics. Jager et al. (2017) further emphasises the scientific advantages of using homogeneous samples in convenience sampling and suggests that the use of homogeneous convenience samples as a positive alternative to traditional convenience samples can improve the internal validity of a study.

The non-probability sampling method of selecting homogeneous samples is an important strategy in theoretical international research design, which is appropriate for the objectives of this study and offers scientific advantages in ensuring the relevance of specific group characteristics and ensuring the internal and external validity of the study. Therefore, to achieve the objectives of this study, the researcher will use convenience sampling in non-probability sampling and select a homogeneous sample to ensure the homogeneity of the sample in terms of key characteristics.

The sale of tea bags is experiencing significant growth in China’s tea beverage industry. It is essential to explore how young consumers respond to the VEPD of tea bags to better understand consumer feedback on tea bag packaging and inform the future development of tea bag brand and product VEPD. Therefore, tea bag product and brand packaging has been selected as the research focus for this study.

Then, the sample characteristics were further determined. According to iiMedia Research (2021), during the period of March 2020 to March 2021, China’s tea bag users were mainly concentrated in North China, with Hebei Province showing the highest proportion of users at 24.64 percent, significantly higher than other provinces. Given that Hebei Province has the highest concentration and most representative population of tea bag users in China, Shijiazhuang City in Hebei Province was selected as the testing location for this study.

To further define the detailed characteristics of the study sample, reports from iiMedia Research (2021) and Ipsos (2020) indicate that people aged 20 to 29 years are the primary consumers of tea bags. Therefore, to ensure a homogeneous sample, i.e., one with a high degree of similarity in key socio-demographic characteristics, this study identifies the current study population as the main consumers of tea bag brands and products in Shijiazhuang City, Hebei Province, China, within the age range of 20 to 29 years.

To ensure sample validity and homogeneity, respondents were required to answer positively to the question ‘Have you ever purchased a tea bag product or brand?’ before proceeding with the questionnaire. Respondents were also required to select their age range, and only those meeting the criteria were permitted to complete the questionnaire. Upon receipt of the completed questionnaires, the researchers reviewed all responses for IP addresses, and those not meeting the predetermined criteria were excluded to maintain the validity and homogeneity of the study sample.

The sample size estimation for the formal research phase of this study followed a two-step process. First, using the method of Krejcie and Morgan (1970), a baseline sample size of 384 was established. Second, considering the minimum sample size requirements for SEM, which suggests a range of 340 to 680 participants, the researchers determined that an appropriate sample size for this study would be between 384 and 500 participants to ensure scientific rigour in the SEM analysis.

After data cleaning and screening, 490 valid responses were retained for analysis. Through a randomisation algorithm, the participants were evenly distributed to evaluate the 14 packaging design prototypes, with 35 participants per prototype. Data collection followed a structured protocol: the participants received a study briefing and provided informed consent before viewing their assigned prototype and completing measurement items in random order. The mean completion time of 252 s indicated sufficient participant attention to the assessment task.

Before questionnaire distribution, this study received approval from the Research Ethics Committee of Universiti Teknologi MARA (UiTM) (Reference No. REC/11/2023 (PG/MR/453)). All methods were carried out in accordance with relevant guidelines and regulations. Informed consent was obtained from all participants following a detailed explanation of the study objectives and protocol. All data were collected anonymously, and participants were informed that they could withdraw from the study at any time without any consequences. By adhering to these ethical principles, the researchers ensured both scientific validity and reliability of the online questionnaire study whilst protecting participants’ rights and interests.

## 4. Results

The formal study was conducted from 12 to 22 March 2024. From a total distribution of 550 questionnaires, 533 valid responses were received, representing a 97 percent response rate. This response rate meets established standards and supports the reliability of results. After further quality screening, excluding responses with completion times under 180 s, 490 valid and usable responses were retained for statistical analysis. A common method bias test was conducted during the data preparation stage to ensure the findings’ reliability.

### 4.1. Common Method Bias

The testing and control of common method bias (CMB) represents an essential step in ensuring the reliability of study results. To effectively control CMB, this study employed procedural strategies and statistical methods to address common CMB concerns (Cham et al., 2021; Chen et al., 2021; MacKenzie & Podsakoff, 2012; Podsakoff et al., 2024).

The procedural strategies employed in this study encompassed several key measures to ensure methodological rigour. The scale items were sourced from well-established scales across different sources, and the questionnaire was structured with a balanced number of questions to prevent respondent fatigue. The research design carefully avoided complexity, abstraction, and double-barrelled questions, whilst ensuring no conceptual overlap existed between questions in different structures.

To maintain data integrity, complete anonymity was guaranteed during the questionnaire collection phase. Prior to the formal study, a pre-study was conducted using a sample from the target respondent group. This preliminary phase served two crucial purposes: it eliminated potential ambiguities in the instructions and helped to refine the terminology used in the questionnaire. The formal study commenced only after incorporating the insights gained from this pre-study phase, ensuring clarity in both questionnaire description and terminology.

In addition to the procedural strategies, this study employed statistical testing methods. The primary approach utilised was Harman’s (1976) one-factor test, which is the most widely adopted method for assessing CMB in academic research (Fuller et al., 2016; Kumar Mishra et al., 2016). This method examines whether a single factor accounts for the majority of variance by incorporating all measurement items in an exploratory factor analysis without factor rotation.

The Harman one-factor test, conducted after data collection, determines whether a single factor is responsible for the data variance (Chang et al., 2010). The results from the exploratory factor analysis revealed that the first factor explained 35.163 percent of the total variance, falling below the maximum threshold of 50 percent (Malhotra et al., 2017). This finding suggests that CMB does not significantly influence the current dataset.

### 4.2. Descriptive Statistical Analysis

This study analysed the descriptive statistics from 490 valid questionnaires using SPSS (version 29.0) software. Table 3 presents the demographic characteristics of the sample, including age, gender, education level, monthly income, and tea bag purchasing experience.

The results confirmed that all respondents fell within the target age group of 20 to 29 years and had previous tea bag purchasing experience, thus fully conforming to the predetermined sample characteristics. The gender distribution showed 47.1 percent male and 52.9 percent female respondents, indicating a slightly higher female representation.

Regarding educational attainment, which ranged from junior high school to postgraduate level and above, the majority of respondents held undergraduate degrees (54.9%), followed by university junior college (25.3%), with 14.1 percent reporting postgraduate qualifications or higher.

Monthly income analysis revealed that the majority of respondents (57.6%) earned between CNY 4000 and CNY 5999, whilst approximately one-sixth (14.5%) reported monthly earnings between CNY 2000 and CNY 3999.

### 4.3. Measurement Model Evaluation

The relationship between variables and their corresponding indicators is reflected through measurement models, whilst structural models demonstrate the relationships between variables (Wee & Choong, 2019). Analysing measurement models serves as an essential prerequisite for testing research structural equation models to examine the paths of influence between variables (Khowjoy et al., 2023).

This study’s measurement model assessment encompasses several key aspects: constitutive reliability (CR), convergent validity, discriminant validity, and model fit indices. The detailed results of this measurement model evaluation will be presented systematically in the following subsections.

#### 4.3.1. Component Reliability and Convergent Validity

The researchers analysed 490 valid questionnaires using Mplus (version 8.3) software to assess CR and convergent validity. Confirmatory factor analysis using the maximum likelihood method was employed to validate the reliability and validity of the study’s constructs. This assessment ensured the internal consistency and reliability of the measurement scales.

Standardised factor loadings below 0.5 typically require deletion (Bagozzi & Yi, 2012), with values of 0.7 or above being preferable (Bollen, 2020). As shown in Table 4, all item loadings ranged from 0.646 to 0.889, indicating acceptable results. Given the limitations of Cronbach’s alpha in SEM, this study assessed both Cronbach’s alpha and CR values (Gelhard & Von Delft, 2015).

The recommended thresholds for CR and average variance extracted (AVE) are 0.7 and 0.5, respectively (Hair, 2010). The measurement model metrics presented in Table 4 demonstrate Cronbach’s alpha values exceeding 0.7 for all constructs, indicating good sample reliability (Nunnally, 1978). CR scores ranged from 0.764 to 0.934, whilst AVE scores ranged from 0.504 to 0.779, both exceeding their recommended thresholds of 0.7 and 0.5, respectively.

These results confirm that the measurement model demonstrates appropriate CR and convergent validity across all latent variables, meeting the criteria for further SEM analysis.

#### 4.3.2. Discriminant Validity

This study employed two established methods to assess discriminant validity, which evaluates the extent to which constructs in a model are uniquely distinct from one another (Hair et al., 2011). This assessment is crucial in SEM analyses to ensure different variables represent distinct concepts.

The first assessment followed Fornell and Larcker’s (1981) criterion, comparing the square root of AVE for each construct with its correlations to other constructs. Table 5 and Table 6 present the correlation coefficient matrices for lower-order and higher-order constructs, respectively, with diagonal elements showing the square roots of AVE. The analysis revealed that the square roots of AVE in the diagonal portions exceeded the correlations between constructs in off-diagonal entries, indicating satisfactory discriminant validity between latent variables (Fornell & Larcker, 1981).

The second assessment utilised the Heterotrait–Monotrait Ratio (HTMT) proposed by Henseler et al. (2015), calculating the ratio of heterogeneous to homogeneous trait correlations for each construct. The academic literature suggests that HTMT values should not exceed 0.85 or 0.90 in order to demonstrate discriminant validity (Franke & Sarstedt, 2019; Henseler et al., 2015; Voorhees et al., 2016). Table 7 presents the HTMT values, Tolerance values, and Variance Inflation Factor (VIF) values. The results showed all measurement constructs maintained HTMT values below 0.85, Tolerance values above 0.1, and VIF values below five, confirming no discriminant validity concerns (Henseler et al., 2015).

These comprehensive assessments demonstrate that all latent variables in the measurement model possess sufficient discriminant validity and show no multicollinearity issues, making them suitable for further SEM analyses.

#### 4.3.3. Model Fit Indices

This study employed Mplus (version 8.3) software to assess model fit using data from 490 valid questionnaires. Several key indices were examined to evaluate the model’s structural alignment with the data.

The chi-square value serves as a primary indicator of model fit, with a non-significant value suggesting model acceptability. Typically reported as a general index, the ratio of chi-square value to degrees of freedom should preferably be less than three, though values up to five may be acceptable in certain cases (Kline, 2004).

The Comparative Fit Index (CFI) evaluates model fit improvement by considering model complexity and comparing the proposed model with a null model that assumes no variable correlations. The Tucker–Lewis Index (TLI) similarly accounts for model complexity and parameter numbers, with values exceeding 0.90 considered acceptable and those above 0.95 indicating good fit.

The Root Mean Square Error of Approximation (RMSEA) assesses model error approximation whilst accounting for model complexity and sample size. RMSEA values below 0.05 indicate good fit, whilst values between 0.05 and 0.08 suggest acceptable fit (Browne & Cudeck, 1992).

The Standardised Root Mean Square Residual (SRMR) represents the standardised mean difference between observed and model-predicted correlation coefficients. Lower values indicate better fit, with values below 0.08 typically considered good (Hu & Bentler, 1999).

As presented in Table 8, the results align with these established fit indices, collectively demonstrating that the theoretical model adequately fits the observed data. This confirmation of measurement model fit provides a solid foundation for subsequent hypothesis testing.

### 4.4. Structural Model Analysis

Having confirmed the satisfactory reliability and validity indicators in the measurement model, this study proceeded to evaluate the structural model. Structural models serve as a key component in SEM, representing theory-based relationships between constructs (Byrne, 2010; Kelloway, 2015).

This evaluation phase aimed to test the study’s hypotheses and validate the proposed theoretical framework. The researchers conducted SEM analyses using Mplus (version 8.3) software to examine both direct path and mediation effects. The detailed results of these analyses are presented in the subsequent subsections.

#### 4.4.1. Path Hypothesis Testing

The SEM path analysis results, presented in Table 9, reveal significant relationships between packaging design colour elements and PI. The first level of colour element, monochromatic harmony, demonstrated a significant positive effect on PI (β = 0.254, *p* = 0.000) relative to the average of all levels. Similarly, the second level, analogous harmony, showed a significant positive effect (β = 0.192, *p* = 0.004) compared to the level average. Conversely, the third level of colour element, contrast harmony, exhibited a significant negative effect on PI (β = −0.446, *p* ≈ 0.000) when compared to the average of all levels.

The analysis of graphics elements’ effects on PI yielded varied results. The first level of graphics element, figurative, demonstrated a significant positive effect on PI (β = 0.153, *p* = 0.009) relative to the average of all levels. In contrast, the second level, abstract, showed a significant negative effect (β = −0.095, *p* = 0.021) compared to the level average. The third level of graphics element, hybrid, did not demonstrate a statistically significant effect on PI (β = −0.058, *p* ≈ 0.473) when compared to the average of all levels.

The analysis revealed that none of the logo elements demonstrated statistically significant effects on PI. The first level, logomark, showed no significant effect relative to the average of all levels (β = 0.085, *p* = 0.079). Similarly, the second level, logotype, demonstrated no significant effect (β = 0.000, *p* = 0.993) compared to the level average. The third level, combination mark, also failed to show a statistically significant effect on PI (β = −0.085, *p* ≈ 0.206) when compared to the average of all levels.

The analysis of typography elements revealed varying effects on PI across different typeface styles. The first level, Chinese character print typeface, showed no statistically significant influence on PI (β = 0.013, *p* = 0.811) relative to the average of all levels. The second level, calligraphy typeface, demonstrated a significant positive effect (β = 0.126, *p* = 0.019) compared to the level average. The third level, artistic typeface, did not reach statistical significance in its effect on PI (β = −0.139, *p* ≈ 0.063) when compared to the average of all levels.

The analysis of layout elements revealed diverse effects on PI across different design arrangements. The first level, bilateral symmetry, demonstrated a significant positive effect on PI (β = 0.127, *p* = 0.010) relative to the average of all levels. In contrast, both the second level, grid (β = −0.123, *p* = 0.007), and the third level, centralised (β = −0.164, *p* = 0.000), showed significant negative effects compared to the level average. The fourth level, diagonal layout, did not reach statistical significance in its effect on PI (β = 0.160, *p* ≈ 0.053) when compared to the average of all levels.

#### 4.4.2. Mediation Effect Testing

Based on our conceptual model, we constructed the model using BE as a mediating variable. We calculated direct, indirect, and total effects following established procedures (Hayes, 2022; Kline & Little, 2023; J. Wang & Wang, 2020). Direct effects were estimated as standardised path coefficients in the structural model. Indirect effects were calculated as the product of path coefficients (a × b) connecting VEPD to PI through BE. The total effect comprised the sum of direct and indirect effects.

We employed the bootstrap method to estimate the mediating effect. While the Sobel test is common, it can lack accuracy with small samples or non-normal distributions. The bootstrap method provides more precise confidence interval estimates (Hair et al., 2017; Taylor et al., 2008), particularly when the statistic’s behaviour is ambiguous, complex, or highly dependent on specific sampling scenarios (Hayes, 2022). We conducted 5000 replicate samples to estimate BE’s mediating effect value and its 95 percent confidence interval. A confidence interval excluding zero indicated statistical significance in the mediation effect.

Table 10 presents the direct, indirect, and total effects of the overall mediation model. The analysis of the VEPD revealed a significant direct effect on PI, with an estimated value of 0.229 and a 95 percent confidence interval of [0.097, 0.413]. As this confidence interval excludes zero, it confirms the significance of the direct effect.

The indirect effect through BE demonstrated an estimated value of 0.844, with a 95 percent confidence interval of [0.527, 1.205]. The exclusion of zero from this confidence interval establishes statistical significance, confirming that BE serves as a significant mediator in the relationship between packaging design visual elements and PI.

The analysis revealed both significant direct and indirect effects, indicating partial mediation in the relationship between VEPD and PI. The total effect of VEPD on PI showed an estimated value of 1.073, with a 95 percent confidence interval of [0.759, 1.399]. The exclusion of zero from this interval confirms the overall significant effect of VEPD on PI.

These findings support H2, validating the inclusion of BE in the theoretical model. Specifically, the results demonstrate that VEPD influences PI both directly and indirectly through BE, confirming the partial mediating role of BE in this relationship.

As shown in Table 11, the analysis of colour elements revealed significant direct and indirect effects through BE. The direct effect of colour elements on PI showed an estimated value of 0.461, with a 95 percent confidence interval of [0.288, 0.617]. The exclusion of zero from this interval confirms the significance of the direct effect, supporting H1a.

**Figure 3:**
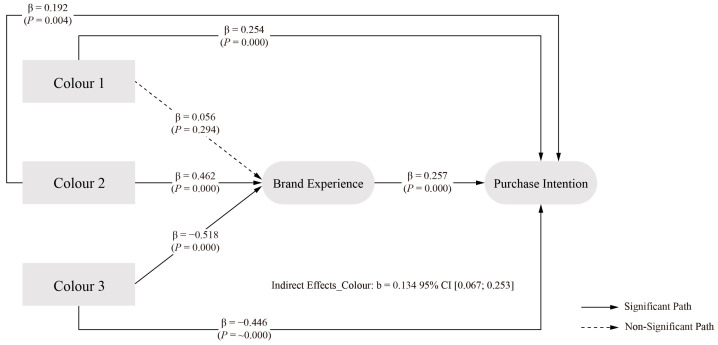
Final model with standardised effects, 95% cis, and *p*-values for various levels of colour elements. Source: author’s drawing.

The indirect effect through BE demonstrated an estimated value of 0.134, with a 95 percent confidence interval of [0.067, 0.253]. As this confidence interval excludes zero, it establishes the significance of BE as a mediator in the relationship between colour elements and PI. Given the significance of both direct and indirect effects, the results indicate partial mediation, with colour elements influencing PI through both pathways. The total effect of colour elements on PI showed an estimated value of 0.595, with a 95 percent confidence interval of [0.455, 0.753]. The exclusion of zero from this interval confirms the overall significant effect of colour elements, with the indirect benefit representing a substantial proportion of the total effect, as indicated by the significant R value. These findings support H2a, and the final model of packaging design colour elements at different levels is illustrated in Figure 3.

The analysis of graphics elements revealed a different pattern of mediation effects compared to colour elements. The direct effect of graphics elements on PI showed an estimated value of 0.052, with a 95 percent confidence interval of [−0.036, 0.151]. As this interval contains zero, it indicates no significant direct effect, failing to support H1b.

**Figure 4:**
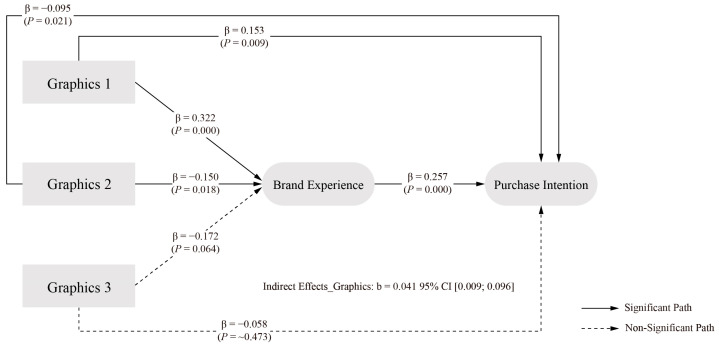
Final model with standardised effects, 95% cis, and *p*-values for various levels of graphics elements. Source: author’s drawing.

The indirect effect through BE demonstrated an estimated value of 0.041, with a 95 percent confidence interval of [0.009, 0.096]. The exclusion of zero from this interval establishes the significance of the indirect effect, confirming BE as a significant mediator in the relationship between graphics elements and PI. The total effect of graphics elements on PI showed an estimated value of 0.093, with a 95 percent confidence interval of [0.003, 0.177]. The exclusion of zero from this interval confirms the overall significant effect. Given the non-significant direct effect but significant indirect effect, the results indicate full mediation, suggesting that graphics elements influence PI entirely through BE. These findings support H2b, and the final model of packaging design graphics elements at different levels is illustrated in Figure 4.

The analysis of logo elements revealed a complex pattern of mediation effects. The direct effect of logo elements on PI showed an estimated value of 0.085, with a 95 percent confidence interval of [−0.005, 0.181]. As this interval contains zero, it indicates no significant direct effect, failing to support H1c.

**Figure 5:**
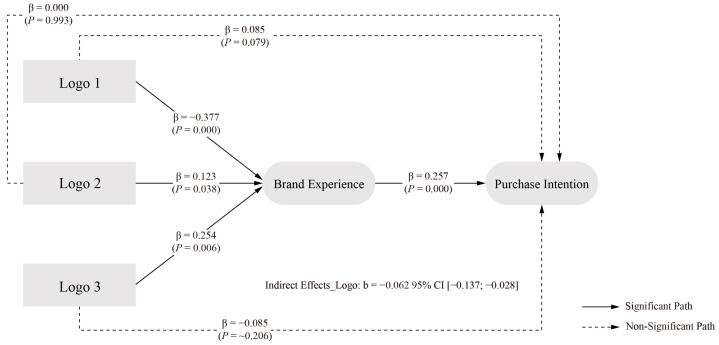
Final model with standardised effects, 95% cis, and *p*-values for various levels of logo elements. Source: author’s drawing.

The indirect effect through BE demonstrated an estimated value of −0.062, with a 95 percent confidence interval of [−0.137, −0.028]. The exclusion of zero from this interval confirms a significant negative indirect effect, establishing BE as a significant mediator in the relationship between logo elements and PI. The total effect of logo elements on PI showed an estimated value of 0.023, with a 95 percent confidence interval of [−0.070, 0.107]. The inclusion of zero in this interval indicates no significant total effect. However, the significant R value suggests that the indirect path through BE may have been counterbalanced by unmeasured influence paths, resulting in the non-significant total effect. This finding underscores the importance of examining mediating mechanisms beyond total effects. These results support H2c, highlighting the crucial role of BE in mediating the relationship between packaging design logo elements and PI. The final model of logo elements at different levels is illustrated in Figure 5.

The analysis of typography elements revealed a distinct pattern of effects compared to other design elements. The direct effect of typography elements on PI showed an estimated value of 0.143, with a 95 percent confidence interval of [0.056, 0.223]. The exclusion of zero from this interval confirms a significant direct effect, supporting H1d.

**Figure 6:**
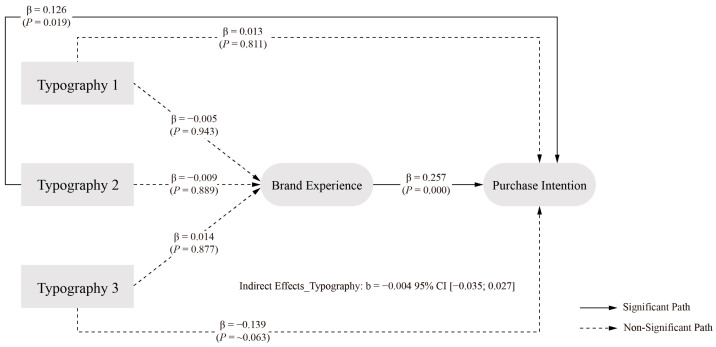
Final model with standardised effects, 95% cis, and *p*-values for various levels of typography elements. Source: author’s drawing.

The indirect effect through BE demonstrated an estimated value of −0.004, with a 95 percent confidence interval of [−0.035, 0.027]. As this interval contains zero, it indicates no significant indirect effect through BE. The total effect of typography elements on PI showed an estimated value of 0.140, with a 95 percent confidence interval of [0.062, 0.221]. The exclusion of zero from this interval confirms a significant overall effect. These results indicate that typography elements influence PI primarily through the direct path, with no significant mediating effect through BE. These findings do not support H2d, suggesting that BE does not serve as a mediator in the relationship between typography elements and PI. The final model of typography elements at different levels is illustrated in Figure 6.

The analysis of layout elements revealed significant effects through both direct and indirect pathways. The direct effect of layout elements on PI showed an estimated value of −0.201, with a 95 percent confidence interval of [−0.321, −0.077]. The exclusion of zero from this interval confirms a significant negative direct effect, supporting H1e.

**Figure 7:**
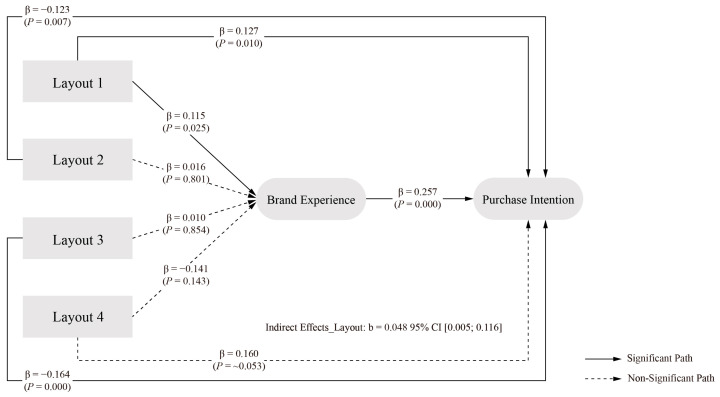
Final model with standardised effects, 95% cis, and *p*-values for various levels of layout elements. Source: author’s drawing.

The indirect effect through BE demonstrated an estimated value of 0.048, with a 95 percent confidence interval of [0.005, 0.116]. The exclusion of zero from this interval establishes the significance of the indirect effect, confirming BE as a significant mediator in the relationship between layout elements and PI. The total effect of layout elements on PI showed an estimated value of −0.153, with a 95 percent confidence interval of [−0.269, −0.039]. The exclusion of zero from this interval confirms a significant overall effect. The significant R value indicates that the indirect benefit represents a substantial proportion of the total effect. Given the significance of both direct and indirect effects, the results demonstrate partial mediation, with layout elements influencing PI through both pathways. These findings support H2e, and the final model of packaging design layout elements at different levels is illustrated in Figure 7.

## 5. Discussion

This study verified two elements: the direct effect of VEPD on tea bag consumers’ PI and BE’s mediating effect on the relationship between VEPD and PI. This study found evidence that different visual elements (colour, graphics, logo, typography, and layout) in packaging design have different effects on consumers’ PI at different levels. Furthermore, it demonstrated that VEPD influences PI through BE mediation, enriching the understanding of the mechanism between packaging visual attributes and PI.

Part I tested VEPD’s direct effect on tea bag consumers’ PI. Empirical analyses support hypothesis H1, deepening our understanding of how VEPD influences consumer responses. This finding broadly supports other research work in the field linking packaging visual attributes to consumers’ PI (El Oraiby & Kiygi-Calli, 2023; Felix et al., 2022; Fenko et al., 2018; Hess & Melnyk, 2016; Qiao & Griffin, 2022; Schuch et al., 2019; Srivastava et al., 2022). Importantly, this study contributes additional insights by examining different levels of visual design elements, specifically in tea bag packaging.

Regarding colour elements, our results show a significant effect on PI, supporting hypothesis H1a. Specifically, monochromatic harmony (β = 0.254, *p* = 0.000) and analogous harmony (β = 0.192, *p* = 0.004) showed positive effects, while contrast harmony showed a negative effect (β = −0.446, *p* = 0.000). These findings differ from those of Hurley et al. (2017), who found no significant differences between different colour harmonies. The stronger effect of monochromatic harmony is consistent with the empirical findings of Deng et al. (2010), suggesting that consumers prefer similar or identical colours, supporting visual consistency theory rather than optimal arousal.

Regarding typography elements, our analyses show a significant effect on PI, supporting hypothesis H1d. Notably, calligraphy typeface showed a positive effect (β = 0.126, *p* = 0.019), while Chinese character print typeface had no significant effect. These findings contrast with Western studies favouring print typefaces (El Oraiby & Kiygi-Calli, 2023; Kovačević et al., 2022), highlighting potential cross-cultural differences in font preference. The positive impact of calligraphic fonts supports Huang et al.’s (2023) findings of higher price perceptions of calligraphic designs among Chinese consumers.

For layout elements, our analyses showed a significant effect on PI, providing systematic support for hypothesis H1e. Bilateral symmetry showed a positive effect (β = 0.127, *p* = 0.010), while grid and centralised showed a negative effect. These results confirm the importance of packing layout (Silayoi & Speece, 2007; Wen, 2024; Zafar et al., 2022; Zhao et al., 2024) and support the findings of Lacoste-Badie et al. (2020) on the effect of symmetry on visual attention. The negative impact of centralised layout suggests that a single format may not be effective in attracting consumer attention, which is consistent with Yung’s (2023) emphasis on innovative and attractive packaging design.

Interestingly, while graphics did not show a significant overall effect, i.e., the findings failed to provide statistical support for hypothesis H1b, figurative elements showed a positive effect (β = 0.153, *p* = 0.009), supporting Ampuero and Vila’s (2006) earlier findings on the effectiveness of figurative elements. Similarly, logo elements did not show a significant direct effect, i.e., the findings failed to provide statistical support for hypothesis H1c, suggesting a possible indirect effect through the BE, which is discussed further below.

In addition to the direct effects described above, our study also tested the mediating effect of BE in the relationship between VEPD and consumer PI in the second part of the study, and the results of the empirical analyses supported hypothesis H2. This finding broadly supports other work in the field that has used BE as a mediating variable linking a range of brand touchpoints to consumers’ PI (Gao & Shen, 2024; Khan & Rahman, 2015; Rather et al., 2024; Venter de Villiers et al., 2018). Importantly, by focusing on the VEPD of tea bag, our study extends previous research on the mechanisms of BE (Bigoin-Gagnan & Lacoste-Badie, 2018; Chen et al., 2021; Moreira et al., 2017; Nivedhitha & Manzoor, 2020; Qi & Yan, 2020; Venter de Villiers et al., 2018; Yasri et al., 2020).

Our findings emphasise that specific visual packaging elements can enhance consumers’ BE, which in turn promotes PI. This reinforces the role of packaging as a brand-relevant stimulus (Brakus et al., 2009; Schmitt, 1999) and as a key medium for brand–consumer communication (Hassan, 2018; Heiltjes, 2014; Krishna et al., 2017; Rundh, 2009; Titah, 2022; E. S.-T. Wang, 2017). Visual elements establish sensory, affective, and cognitive connections with consumers (Shukla et al., 2022), thus enhancing BE.

By examining specific visual elements, we found that the colour element showed a partial mediation effect (indirect effect = 0.134, 95% CI [0.067, 0.253]), i.e., hypothesis H2a holds, supporting the research on how package colours can influence consumer decision-making through various pathways (Kauppinen-Räisänen, 2014; Labrecque & Milne, 2012; Wei et al., 2014; J. Su & Wang, 2024). The graphics element showed a fully mediated effect (indirect effect = 0.041, 95% CI [0.009, 0.096]), i.e., hypothesis H2b holds, confirming Orth and Malkewitz’s (2008) findings regarding the systematic effect of packaging on perceived brand personality.

The logo element showed a significant indirect effect (indirect effect = −0.062, 95% CI [−0.137, −0.028]), i.e., hypothesis H2c holds, supporting the argument for the role of a logo in enhancing positive brand attitudes (Septianto & Paramita, 2021) The layout element also showed a partial mediating effect (indirect effect = 0.048, 95% CI [0.005, 0.116]), i.e., hypothesis H2e holds, in line with studies emphasising the impact of layout on consumer experience (Oyibo & Vassileva, 2020; Tang et al., 2024; Williamson, 2019). Interestingly, typography elements did not show significant indirect effects through BE (95% CI [−0.035, 0.027]), i.e., hypothesis H2d did not hold, suggesting that their effects may be more direct than mediated effects.

## 6. Conclusions

### 6.1. Theoretical Implications

This study develops a conceptual model grounded in S-O-R theory, visual design elements theory, and brand experience model theory, integrating design and marketing perspectives to examine the relationships between VEPD, BE, and PI. Through an empirical investigation of tangible VEPD as brand touchpoints, the research reveals significant insights into consumer behaviour and decision-making processes. The findings demonstrate that VEPD significantly influences PI through both direct and indirect pathways, establishing a partial mediation effect. Specifically, the research illuminates how different levels of visual attributes in tea bag packaging affect consumer responses. This granular analysis enhances the current understanding of the relationship between VEPD and PI.

Moreover, this study addresses a critical gap in existing research by examining the specific mechanisms through which design elements influence PI, viewed through the lens of BE. This novel approach provides valuable theoretical contributions to the field, which will be detailed in subsequent sections.

This study makes significant theoretical contributions through its application of S-O-R theory. By employing this theory as a fundamental framework, the research validates the mechanisms through which packaging design elements influence consumer behaviour, whilst expanding the theoretical framework’s scope of application. The study’s conceptual framework positions VEPD as an external stimulus that affects consumers’ internal mediating constructs (BE), which subsequently influence behavioural responses (PI). This structured approach provides valuable insights for companies seeking to optimise their packaging strategies for enhanced market performance. The innovative integration of visual design elements theory and brand experience model theory with S-O-R theory offers a novel analytical framework for understanding tea bag packaging. This multifaceted theoretical approach not only broadens existing research perspectives but also establishes a more comprehensive framework for analysing packaging design’s impact on consumer behaviour.

The integration of multiple theoretical frameworks in this study represents a significant advancement in consumer behaviour research. While brand experience model theory is widely recognised in consumer behaviour studies, its incorporation within the S-O-R theoretical framework has been limited. This study pioneers the novel integration of brand experience model theory and visual design elements theory within the S-O-R framework, a combination rarely explored in the existing literature. This theoretical synthesis enhances the generalisability of S-O-R theory while extending both visual design elements theory and brand experience theory. The research provides a detailed analysis of individual visual elements (colour, graphics, and typography) across different levels and design forms, offering comprehensive insights into their contributions to brand perception and consumer behaviour.

The study’s examination of specific VEPD characteristics and their influence mechanisms provides a crucial understanding of how and when particular design features affect consumer behaviour. This granular analysis advances design theory by offering more precise insights into the impact of visual elements, while simultaneously providing empirically grounded guidance for design practitioners. This enhanced understanding enables the more accurate prediction and application of design elements’ effects on consumer responses, bridging the gap between theoretical knowledge and practical implementation.

In summary, this study advances the understanding of visual design elements’ influence mechanisms by integrating a multidisciplinary theoretical framework within the context of low-involvement products. Through the construction of this comprehensive framework, the research reveals specific mechanisms of action for each sub-element, providing scientifically grounded guidance for marketing practice. The findings extend current knowledge regarding the relationships between VEPD, BE, and PI, particularly within low-involvement contexts, whilst offering novel theoretical perspectives on consumer purchase decision-making based on visual design elements theory.

The research addresses significant theoretical gaps in the existing literature and creates new pathways for understanding consumer purchase decisions. These insights prove valuable not only for future research directions but also for brands and companies seeking to optimise their product visual design to enhance consumer BE and PI. The study’s multidisciplinary approach and detailed examination of specific design elements provide a robust foundation for both theoretical advancement and practical application in marketing strategy.

### 6.2. Practical Implications

The empirical results of this study reveal a complex relationship between VEPD, BE, and PI, and these findings have important implications for designers and marketing practitioners. By analysing the impact of VEPD on PI, this study strongly suggests that practitioners should pay attention to the use of VEPD, especially colour, typography, and layout. Specifically, the design of colour visual elements should consider the use of monochromatic harmony and analogous harmony colour schemes, and the appropriate use of contrast harmony, as an overly contrasting colour scheme may lead to negative evaluations by consumers. For typography VEPD, the use of calligraphy typeface can be considered. It is important to focus on calligraphy typeface in the design of bagged tea packaging to attract Chinese consumers and to meet their expectations and preferences. For the design of layout, it is recommended to consider the bilateral symmetry layout, which is considered to be more attractive, and the rational use of bilateral symmetry can effectively enhance the PI of consumers. Therefore, this study suggests that practitioners should pay attention to the application of VEPD, especially colour, typography, and layout elements, which have a beneficial effect on consumers’ PI.

In addition, by analysing the mediating role of BE between VEPD and PI, specific mechanisms of influence between each visual element and purchase decision were identified. This study strongly suggests that practitioners should pay attention to the BE of consumers, especially in terms of the colour, graphics, logo, and layout elements. It is suggested that by developing BE strategies for consumers, it is possible to facilitate more positive PI. Specifically, for the colour visual element, the adoption of an analogous harmony colour scheme to effectively enhance the BE for consumers should be considered, as should the forming of a harmonious analogous colour scheme. For the design of graphics visual elements, priority should be given to figurative style graphics, which are more effective in evoking emotional and cognitive connections among consumers. For the VEPD of a logo, the combination mark can effectively enhance the BE of consumers. This kind of logo combines non-textual graphics with textual expressions, and the two complement each other and rely on each other to play a role in each other. In the current competitive market, especially for low-involvement product packaging design, combination mark is able to combine the advantages of graphics and text, which is more outstanding in attracting consumers’ attention and creating brand identity. Bilateral symmetry should be considered for the layout of visual elements to enhance the BE for consumers. The above recommendations aim to optimise and build a more comprehensive BE system. Therefore, this study suggests that practitioners should pay attention to consumer BE, especially colour, graphics, logo, and layout elements, which have an impact on consumer BE and PI.

### 6.3. Limitations and Future Research

Although this study provides valuable insights and practical implications, it is important to consider certain limitations, which in turn provide interesting avenues for future research. Firstly, in terms of cultural context, this study focuses on the Chinese context, so the exploration of typography elements is limited to the Chinese typography of Chinese characters. The results of the study not only validate the influence of typography elements on consumers’ PI but also further clarify the specific mechanism of the influence of different levels of typography on consumers. The use of calligraphy typeface, compared with Chinese character print typeface and artistic typeface in the packaging design of tea bags, has a significant positive effect on consumers’ purchasing decisions, which provides designers and marketers with more accurate design strategies and a scientific basis.

Interestingly, considering the similarity between Western typography and Chinese typography to a certain extent, future research could replace the levels of packaging design typography elements with the typography type of the country in the specific research context (e.g., different levels of serif, sans-serif and script (Vladimirova, 2017)). It is also possible to consider the inclusion of country-specific cultural dimensions as moderators in the model of this study, which would provide additional insights into the field of research on the relationship between VEPD, BE, and PI.

Secondly, this study uses non-probability sampling technique. Considering that non-probability sampling may limit the generalisability of the findings to a certain extent, this study focuses on homogeneous samples, i.e., specific groups with experience in purchasing bagged tea in a specific age group in the selection of the research sample, especially the young consumer group of bagged tea. This ensures the internal and external validity of the study, allows for effective insights in a specific group, and will have higher relevance and applicability to the target group, thus reducing to a certain extent the research bias due to the problem of representativeness of the sample population. In addition, whilst data collected from a single source may be subject to CMB, a range of procedural strategies and statistical methods have been used in this study to effectively control for CMB to ensure that this study is free from significant common methodological bias. Although this study has focused on a specific sample group and used a range of effective measures to control for CMB, further research could be conducted to validate the results or add to the findings in conjunction with other data sources or research methods.

Finally, the generalisability of the findings in the tested industry is also somewhat limited. The selection of packaging categories in this study focuses on bagged tea products in the food industry, and although it provides a new perspective for the field, it cannot be said that this result is applicable to all product categories. As food packaging is a low-involvement product, consumer purchasing decisions are more dependent on the influence of visual attributes of the packaging. Therefore, the visual attributes of food packaging are a key factor influencing consumer purchasing behaviour, and it is important to study the visual appeal of low-involvement products to consumers (Behe et al., 2015; Silayoi & Speece, 2004). Future research could conduct similar studies on different food category packaging to better understand possible cultural differences and broader research perspectives, or go beyond the food industry and investigate other low-involvement products such as cosmetics and daily necessities. It would be interesting to validate whether the results show similar patterns for the broader food industry or for other low-involvement products as a way of expanding the study’s adaptation.

## Figures and Tables

**Table 1:** Factors and levels of visual elements of packaging design.

Factor	Level
Colour	Monochromatic Harmony, Analogous Harmony, Contrast Harmony
Graphics	Figurative, Abstract, Hybrid
Logo	Logomark, Logotype, Combination Mark
Typography	Chinese Character Print Typeface, Calligraphy Typeface, Artistic Typeface
Layout	Bilateral Symmetry, Grid, Centralised, Diagonal

**Table 2:** The final mixed orthogonal table of this study.

Case	Level				
1	A1	B1	C1	D1	E1
2	A2	B2	C2	D2	E1
3	A3	B3	C3	D3	E1
4	A1	B2	C3	D1	E2
5	A2	B1	C1	D3	E2
6	A3	B1	C2	D2	E2
7	A1	B3	C2	D3	E3
8	A2	B2	C1	D1	E3
9	A3	B1	C3	D2	E3
10	A3	B2	C1	D3	E4
11	A2	B3	C2	D1	E4
12	A1	B3	C1	D2	E4
13	A2	B1	C3	D3	E4
14	A3	B3	C1	D1	E2

Note: A1 = monochromatic harmony, A2 = analogous harmony, A3 = contrast harmony; B1 = figurative, B2 = abstract, B3 = hybrid; C1 = logomark, C2 = logotype, C3 = combination mark; D1 = Chinese character print typeface, D2 = calligraphy typeface, D3 = artistic typeface; E1 = bilateral symmetry, E2 = grid, E3 = centralised, E4 = diagonal.

**Table 3:** Demographic characteristics of the sample (*n* = 490).

Variable	Categories	Frequency (Number of People)	Percent (%)
Age (Years)	Under 20 years old (not including 20 years old)	0.0	0.0
	20–29 years old	490	100.0
	Over 29 years old (not including 29 years old)	0.0	0.0
Tea Bag Buying Experience	Have not purchased	0.0	0.0
	Purchased	490	100.0
Gender	Male	231	47.1
	Female	259	52.9
Education Level	Junior high school and below	5	1.0
	High school/technical secondary school/technical school	23	4.7
	University junior college	124	25.3
	Undergraduate	269	54.9
	Graduate students and above	69	14.1
Monthly Income (CNY)	No income	49	10.0
	Less than CNY 2000 (not including CNY 2000)	32	6.5
	CNY 2000–3999	71	14.5
	CNY 4000–5999	282	57.6
	CNY 6000–7999	42	8.6
	CNY 8000–10,000	10	2.0
	More than CNY 10,000 (not including CNY 10,000)	4	0.8

Source: SPSS 29.

**Table 4:** Measurement properties (composite reliability and convergent validity results).

Constructs	Items	Significance Estimation			Question Reliability			Cronbach’s Alpha	CR	AVE
		UnStd.	S. E.	Z	*p*-Value	Std.	SMC			
SE	SE1	1.000				0.722	0.521	0.763	0.764	0.518
	SE2	1.027	0.089	11.519	***	0.712	0.508			
	SE3	1.057	0.074	14.280	***	0.726	0.528			
AE	AE1	1.000				0.721	0.521	0.750	0.803	0.505
	AE2	0.936	0.075	12.481	***	0.668	0.446			
	AE3	1.032	0.087	11.803	***	0.733	0.538			
IE	IE1	1.000				0.716	0.513	0.801	0.802	0.504
	IE2	1.009	0.074	13.562	***	0.763	0.582			
	IE3	0.979	0.070	13.949	***	0.709	0.502			
	IE4	0.918	0.078	11.797	***	0.646	0.417			
BE	SE	1.000				0.746	0.557	0.834	0.780	0.542
	AE	1.096	0.137	7.979	***	0.755	0.570			
	IE	1.045	0.127	8.235	***	0.707	0.500			
PI	PI1	1.000				0.873	0.763	0.934	0.934	0.779
	PI2	1.006	0.033	30.841	***	0.889	0.789			
	PI3	1.014	0.036	28.382	***	0.888	0.789			
	PI4	1.059	0.038	27.913	***	0.881	0.777			

Note: UnStd. = unstandardised; S. E. = standard errors of the regression weights; Z = Z-score; Std. = standardised; SMC = square multiple correlations; CR = composite reliability; AVE = average variance extracted; Significance levels: *** denotes *p* < 0.001; SE = sensory experience; AE = affective experience; IE = intellectual experience; BE = brand experience; PI = purchase intention. Source: SPSS 29. Mplus 8.3.

**Table 5:** Correlations matrix (discriminant validity results).

Constructs	CR	AVE	SE	AE	IE	PI
SE	0.764	0.518	**0.720**			
AE	0.803	0.505	0.565	**0.711**		
IE	0.802	0.504	0.516	0.554	**0.710**	
PI	0.934	0.780	0.345	0.309	0.340	**0.883**

Note: Diagonal bold entries are values of the square roots average variance extracted (AVE); all others are correlations coefficients. CR = composite reliability; AVE = average variance extracted; SE = sensory experience; AE = affective experience; IE = intellectual experience; PI = purchase intention. Source: Mplus 8.3.

**Table 6:** Correlations matrix (discriminant validity results).

Constructs	CR	AVE	BE	PI
BE	0.780	0.542	**0.736**	
PI	0.934	0.779	0.450	**0.883**

Note: Diagonal bold entries are values of the square roots average variance extracted (AVE); all others are correlations coefficients. CR = composite reliability; AVE = average variance extracted; BE = brand experience; PI = purchase intention. Source: Mplus 8.3.

**Table 7:** Heterotrait–Monotrait Ratio.

Constructs	SE	AE	IE	PI	BE	Tolerance	VIF
SE						0.735	1.361
AE	0.565					0.728	1.374
IE	0.508	0.546				0.745	1.342
PI	0.347	0.313	0.335			0.658	1.519
BE				0.408		0.696	1.436

Note: Heterotrait–Monotrait Ratio (HTMT) Values < 0.85 indicate good discriminant validity. SE = sensory experience; AE = affective experience; IE = intellectual experience; PI = purchase intention; BE = brand experience; VIF = Variance Inflation Factor. Source: SPSS 29. Mplus 8.3.

**Table 8:** Fit indices for the structural equation model.

Model Fit Indices	Full Name	Value	Recommended Standards	Compliance
χ^2^	Chi-Square Statistic	156.335	Lower values are preferable	Yes
χ^2^/df	Chi-Square to Degrees of Freedom Ratio	1.212	≤3 or ≤5	Yes
CFI	Comparative Fit Index	0.995	>0.90 Good, >0.95 Excellent	Yes
TLI	Tucker–Lewis Index	0.994	>0.90 Good, >0.95 Excellent	Yes
RMSEA	Root Mean Square Error of Approximation	0.021	≤0.05 Good, ≤0.08 Acceptable	Yes
SRMR	Standardised Root Mean Square Residual	0.028	<0.08 Good	Yes

Source: Mplus 8.3.

**Table 9:** Structural equation modelling regression weights.

Hypothesis	Paths	β	UnStd.	S. E.	Z	*p*-Value	Result
H1a	Colour 1 → PI	0.254	0.403	0.076	5.298	0.000	Supported
	Colour 2 → PI	0.192	0.289	0.101	2.869	0.004	Supported
	Colour 3 → PI	−0.446	−0.692	0.126	−5.475	0.000	Supported
H1b	Graphics 1 → PI	0.153	0.225	0.086	2.628	0.009	Supported
	Graphics 2 → PI	−0.095	−0.148	0.064	−2.308	0.021	Supported
	Graphics 3 → PI	−0.058	−0.077	0.107	−0.718	0.473	Not supported
H1c	Logo 1 → PI	0.085	0.127	0.072	1.757	0.079	Not supported
	Logo 2 → PI	0.000	0.001	0.071	0.009	0.993	Not supported
	Logo 3 → PI	−0.085	−0.128	0.101	−1.266	0.206	Not supported
H1d	Typography 1 → PI	0.013	0.019	0.080	0.239	0.811	Not supported
	Typography 2 → PI	0.126	0.196	0.083	2.352	0.019	Supported
	Typography 3 → PI	−0.139	−0.215	0.115	−1.865	0.063	Not supported
H1e	Layout 1 → PI	0.127	0.225	0.087	2.592	0.010	Supported
	Layout 2 → PI	−0.123	−0.202	0.075	−2.714	0.007	Supported
	Layout 3 → PI	−0.164	−0.290	0.075	−3.855	0.000	Supported
	Layout 4 → PI	0.160	0.267	0.137	1.946	0.053	Not supported

Note: β = standardised regression coefficient; UnStd. = unstandardised; S. E. = standard errors of the regression weights; Z = Z-score; PI = purchase intention. Source: SPSS 29. Mplus 8.3.

**Table 10:** Mediation model analysis.

Hypothesis (H2)	Paths	UnStd.	Product of Coefficients		Bia-Corrected 95%		*p*-Value
			S. E.	Z	LLCIs	ULCIs	
Direct Effects	VEPD → PI	0.229	0.081	2.846	0.097	0.413	0.004
Indirect Effects	VEPD → BE → PI	0.844	0.172	4.898	0.527	1.205	0.000
Total Effects	VEPD → PI	1.073	0.164	6.538	0.759	1.399	0.000
R	Indirect Effects/Total Effects	0.214	0.081	2.629	0.092	0.434	0.009

Note: R = ratio of indirect effects; UnStd. = unstandardised; S. E. = standard errors of the regression weights; Z = Z-score; LLCIs = lower levels confidence intervals; ULCIs = upper levels confidence intervals; VEPD = visual elements of packaging design; BE = brand experience; PI = purchase intention; 5000 bootstrap samples. Source: Mplus 8.3.

**Table 11:** Mediation model analysis.

Hypothesis (H2)	Paths	UnStd.	Product of Coefficients		Bia-Corrected 95%		*p*-Value
			S. E.	Z	LLCIs	ULCIs	
Direct Effects	Colour → PI	0.461	0.084	5.461	0.288	0.617	0.000
Indirect Effects	Colour → BE → PI	0.134	0.043	3.140	0.067	0.253	0.002
Total Effects	Colour → PI	0.595	0.078	7.621	0.455	0.753	0.000
R	Indirect Effects/Total Effects	0.226	0.077	2.947	0.108	0.438	0.003
Direct Effects	Graphics → PI	0.052	0.048	1.073	−0.036	0.151	0.283
Indirect Effects	Graphics → BE → PI	0.041	0.022	1.897	0.009	0.096	0.058
Total Effects	Graphics → PI	0.093	0.045	2.065	0.003	0.177	0.039
R	Indirect Effects/Total Effects	0.445	3.776	0.118	0.046	2.999	0.906
Direct Effects	Logo → PI	0.085	0.047	1.815	−0.005	0.181	0.070
Indirect Effects	Logo → BE → PI	−0.062	0.023	−2.653	−0.137	−0.028	0.008
Total Effects	Logo → PI	0.023	0.044	0.523	−0.070	0.107	0.601
R	Indirect Effects/Total Effects	−2.658	42.077	−0.063	−1201.495	−0.646	0.950
Direct Effects	Typography → PI	0.143	0.042	3.411	0.056	0.223	0.001
Indirect Effects	Typography → BE → PI	−0.004	0.016	−0.230	−0.035	0.027	0.818
Total Effects	Typography → PI	0.140	0.040	3.470	0.062	0.221	0.001
R	Indirect Effects/Total Effects	−0.026	0.169	−0.154	−0.301	0.241	0.877
Direct Effects	Layout → PI	−0.201	0.063	−3.199	−0.321	−0.077	0.001
Indirect Effects	Layout → BE → PI	0.048	0.028	1.685	0.005	0.116	0.092
Total Effects	Layout → PI	−0.153	0.061	−2.517	−0.269	−0.039	0.012
R	Indirect Effects/Total Effects	−0.313	7.411	−0.042	−1.631	−0.026	0.966

Note: R = ratio of indirect effects; UnStd. = unstandardised; S. E. = standard errors of the regression weights; Z = Z-score; LLCIs = lower levels confidence intervals; ULCIs = upper levels confidence intervals; BE = brand experience; PI = purchase intention; 5000 bootstrap samples. Source: Mplus 8.3.

## Data Availability

The datasets used in the study are available from the corresponding author on reasonable request.

## Abbreviations

The following abbreviations are used in this manuscript:

AE	Affective Experience
AVE	Average Variance Extracted
BE	Brand Experience
CMB	Common Method Bias
CR	Constitutive Reliability
CFI	Comparative Fit Index
HTMT	Heterotrait–Monotrait Ratio
IE	Intellectual Experience
LLCIs	Lower Levels Confidence Intervals
PI	Purchase Intention
PCCS	Practical Colour Coordinate System
RMSEA	Root Mean Square Error of Approximation
R	Ratio of Indirect Effects
S-O-R	Stimulus–Organism–Response
SE	Sensory Experience
Std.	Standardised
SMC	Square Multiple Correlations
SEM	Structural Equation Modelling
SRMR	Standardised Root Mean Square Residual
S. E.	Standard Errors of The Regression Weights
TLI	Tucker–Lewis Index
UnStd.	Unstandardised
ULCIs	Upper Levels Confidence Intervals
VEPD	Visual Elements of Packaging Design
VIF	Variance Inflation Factor
Z	Z-Score

## Appendix A

**Table A1:** Effect coding for independent variable.

No	Factor	Level	Effect Coding (Recoding)
1	Colour	Monochromatic Harmony	1 = Colour 1; 0 = Colour 2.
		Analogous Harmony	0 = Colour 1; 1 = Colour 2.
		Contrast Harmony	−1 = Colour 1; −1 = Colour 2.
2	Graphics	Figurative	1 = Graphics 1; 0 = Graphics 2.
		Abstract	0 = Graphics 1; 1 = Graphics 2.
		Hybrid	−1 = Graphics 1; −1 = Graphics 2.
3	Logo	Logomark	1 = Logo 1; 0 = Logo 2.
		Logotype	0 = Logo 1; 1 = Logo 2.
		Combination Mark	−1 = Logo 1; −1 = Logo 2.
4	Typography	Chinese Character Print Typeface	1 = Typography 1; 0 = Typography 2.
		Calligraphy Typeface	0 = Typography 1; 1 = Typography 2.
		Artistic Typeface	−1 = Typography 1; −1 = Typography 2.
5	Layout	Bilateral Symmetry	1 = Layout 1; 0 = Layout 2; 0 = Layout 3.
		Grid	0 = Layout 1; 1 = Layout 2; 0 = Layout 3.
		Centralised	0 = Layout 1; 0 = Layout 2; 1 = Layout 3.
		Diagonal	−1 = Layout 1; −1 = Layout 2; −1 = Layout 3.

**Table A2:** Scale items for this study.

No	Constructs	No.	Measurement Items
1	Sensory Experience	SE1	I find this packaging interesting in a sensory (visual) way.
		SE2	This packaging appeals to my senses (visual).
		SE3	This packaging makes a strong impression on my visual sense.
2	Affective Experience	AE1	This packaging induces feelings and sentiments.
		AE2	This packaging tries to put me in a certain mood.
		AE3	This packaging appeals to feelings.
3	Intellectual Experience	IE1	I engage in a lot of thinking when I encounter this packaging.
		IE2	This packaging stimulates my curiosity and problem solving.
		IE3	This packaging makes me think in a special way.
		IE4	This packaging makes me learn new things.
4	Purchase Intention	PI1	It is very likely that I will buy the product/brand of this packaging.
		PI2	I would purchase the product/brand of this packaging next time.
		PI3	I think about this product/brand of tea bag as a choice when buying in the tea bag.
		PI4	I think of buying this product/brand of tea bag.

## References

[B1-behavsci-15-00181] Alkharusi H. (2012). Categorical variables in regression analysis: A comparison of dummy and effect coding. International Journal of Education.

[B2-behavsci-15-00181] Amer S. M., Elshimy A. A., Abo El Ezz M. E. S. M. (2023). The role of brand experience on brand equity: Mediating effect of authenticity in new luxury fashion brands. Cogent Business & Management.

[B3-behavsci-15-00181] Ampuero O., Vila N. (2006). Consumer perceptions of product packaging. Journal of Consumer Marketing.

[B4-behavsci-15-00181] Antunes I. F. S., Veríssimo J. M. C. (2024). A bibliometric review and content analysis of research trends in sensory marketing. Cogent Business & Management.

[B5-behavsci-15-00181] Asakura N., Zhao Y. (2018). Fundamental problems of composing with colors.

[B6-behavsci-15-00181] Bagozzi R. P., Yi Y. (2012). Specification, evaluation, and interpretation of structural equation models. Journal of the Academy of Marketing Science.

[B7-behavsci-15-00181] Behe B. K., Bae M., Huddleston P. T., Sage L. (2015). The effect of involvement on visual attention and product choice. Journal of Retailing and Consumer Services.

[B8-behavsci-15-00181] Benachenhou S. M., Guerrich B., Moussaoui Z. (2018). The effect of packaging elements on purchase intention: Case study of Algerian customers. Management Science Letters.

[B9-behavsci-15-00181] Bezaz N., Kacha M. (2021). An experimental study of the effect of packaging colour on children’s evaluation of packaging and attitude towards the brand. International Journal of Retail & Distribution Management.

[B10-behavsci-15-00181] Bigoin-Gagnan A., Lacoste-Badie S. (2018). Symmetry influences packaging aesthetic evaluation and purchase intention. International Journal of Retail & Distribution Management.

[B11-behavsci-15-00181] Bollen K. A. (2020). When good loadings go bad: Robustness in factor analysis. Structural Equation Modeling: A Multidisciplinary Journal.

[B12-behavsci-15-00181] Boon H., Bozinovski N. (2019). A Systematic narrative review of the evidence for labeling of natural health products and dietary supplements. The Journal of Alternative and Complementary Medicine.

[B13-behavsci-15-00181] Brakus J. J., Schmitt B. H., Zarantonello L. (2009). Brand experience: What is it? How is it measured? Does it affect loyalty?. Journal of Marketing.

[B14-behavsci-15-00181] Briand Decré G., Cloonan C. (2019). A touch of gloss: Haptic perception of packaging and consumers’ reactions. Journal of Product & Brand Management.

[B15-behavsci-15-00181] Browne M. W., Cudeck R. (1992). Alternative ways of assessing model fit. Sociological Methods & Research.

[B16-behavsci-15-00181] Byrne B. M. (2010). Structural equation modeling with AMOS: Basic concepts, applications, and programming.

[B17-behavsci-15-00181] Calderon-Monge E., Ramírez-Hurtado J. M., Cuesta I. R. (2024). Labeling and consumer purchases. International Journal of Consumer Studies.

[B18-behavsci-15-00181] Casales-Garcia V., De Las Heras A., Luque A., Gonzalez-Abril L. (2024). Sustainable emotional design based on Industry 4.0 for industrial nougat packaging. Sustainability.

[B19-behavsci-15-00181] Celhay F., Masson J., Garcia K., Folcher P., Cohen J. (2017). Package graphic design and innovation: A comparative study of Bordeaux and Barossa wine visual codes. Recherche et Applications En Marketing (English Edition).

[B20-behavsci-15-00181] Cham T. H., Cheng B. L., Low M. P., Cheok J. B. C. (2021). Brand image as the competitive edge for hospitals in medical tourism. European Business Review.

[B21-behavsci-15-00181] Chang S.-J., Van Witteloostuijn A., Eden L. (2010). From the editors: Common method variance in international business research. Journal of International Business Studies.

[B22-behavsci-15-00181] Chen X., Jiao C., Ji R., Li Y. (2021). Examining customer motivation and its impact on customer engagement behavior in social media: The mediating effect of brand experience. SAGE Open.

[B23-behavsci-15-00181] Ck V., Fukey L. N., Wankhar V. (2022). Does packaging affect consumer preference during the purchase of chocolate?. ECS Transactions.

[B24-behavsci-15-00181] Cohen J., Cohen P., West S. G., Aiken L. S. (2015). Applied multiple regression correlation analysis for the behavioral sciences.

[B25-behavsci-15-00181] Cortina-Mercado M. (2017). Effect of packaging design in the purchase decision process: A comparison of generations. Global Journal of Business Research.

[B26-behavsci-15-00181] Deng X., Hui S. K., Hutchinson J. W. (2010). Consumer preferences for color combinations: An empirical analysis of similarity-based color relationships. Journal of Consumer Psychology.

[B27-behavsci-15-00181] Elam K. (2018). Typographic systems.

[B28-behavsci-15-00181] El Oraiby M., Kiygi-Calli M. (2023). The influence of packaging design visual elements on consumers’ purchase intention: A comparison study on organic food and non-food products. Organic Agriculture.

[B29-behavsci-15-00181] Fang K.-T., Liu M.-Q., Qin H., Zhou Y.-D. (2018). Theory and application of uniform experimental designs.

[B30-behavsci-15-00181] Felix R., González E. M., Castaño R., Carrete L., Gretz R. T. (2022). When the green in green packaging backfires: Gender effects and perceived masculinity of environmentally friendly products. International Journal of Consumer Studies.

[B31-behavsci-15-00181] Fenko A., De Vries R., Van Rompay T. (2018). How strong is your coffee? The influence of visual metaphors and textual claims on consumers’ flavor perception and product evaluation. Frontiers in Psychology.

[B32-behavsci-15-00181] Fornell C., Larcker D. F. (1981). Structural equation models with unobservable variables and measurement error: Algebra and statistics. Journal of Marketing Research.

[B33-behavsci-15-00181] Fraley S., Zalewski J., Oom M., Terrien B. (2024). Design of experiments via taguchi methods—Orthogonal arrays.

[B34-behavsci-15-00181] Franke G., Sarstedt M. (2019). Heuristics versus statistics in discriminant validity testing: A comparison of four procedures. Internet Research.

[B35-behavsci-15-00181] Fuller C. M., Simmering M. J., Atinc G., Atinc Y., Babin B. J. (2016). Common methods variance detection in business research. Journal of Business Research.

[B36-behavsci-15-00181] Gao F., Shen Z. (2024). Sensory brand experience and brand loyalty: Mediators and gender differences. Acta Psychologica.

[B37-behavsci-15-00181] Gelhard C., Von Delft S. (2015). The role of strategic and value chain flexibility in achieving sustainability performance: An empirical analysis using conventional and consistent PLS. 2nd International Symposium on Partial Least Squares Path Modeling: The Conference for PLS Users.

[B38-behavsci-15-00181] Gil N. (2018). New survey unveils 7 in 10 consumers agree packaging design can influence purchasing decisions.

[B39-behavsci-15-00181] GlobalData (2024). China packaging market size, analyzing material type, innovations and forecast to 2028 (Nos. GDPK240006CS-ST).

[B40-behavsci-15-00181] Graves B., Merkle E. C. (2021). A note on identification constraints and information criteria in Bayesian latent variable models. Behavior Research Methods.

[B41-behavsci-15-00181] Grohmann B., Giese J. L., Parkman I. D. (2013). Using type font characteristics to communicate brand personality of new brands. Journal of Brand Management.

[B42-behavsci-15-00181] Haase J., Wiedmann K.-P., Labenz F. (2018). Effects of consumer sensory perception on brand performance. Journal of Consumer Marketing.

[B43-behavsci-15-00181] Haberstroh K., Orth U. R., Bouzdine-Chameeva T., Cohen J., Maria Corsi A., Crouch R., De Marchi R. (2018). Through the lens of self-construal: Cross-cultural variation in consumers’ appreciation of harmony in marketing visuals. International Marketing Review.

[B44-behavsci-15-00181] Hair J. F. (2010). Multivariate data analysis.

[B45-behavsci-15-00181] Hair J. F., Hult G. T. M., Ringle C. M., Sarstedt M. (2017). A primer on partial least squares structural equation modeling (PLS-SEM).

[B46-behavsci-15-00181] Hair J. F., Ringle C. M., Sarstedt M. (2011). PLS-SEM: Indeed a silver bullet. Journal of Marketing Theory and Practice.

[B47-behavsci-15-00181] Han H., Lee K.-S., Song H., Lee S., Chua B.-L. (2019). Role of coffeehouse brand experiences (sensory/affective/intellectual/behavioral) in forming patrons’ repurchase intention: Impact of switching costs. Journal of Hospitality and Tourism Insights.

[B48-behavsci-15-00181] Harman G. (1976). Practical reasoning. The Review of Metaphysics.

[B49-behavsci-15-00181] Hassan A. A. E. (2018). The importance of packaging design as a branding factor in consumer behavior. Fifth International Conference of the Applied Arts Helwan University.

[B50-behavsci-15-00181] Hayes A. F. (2022). Introduction to mediation, moderation, and conditional process analysis: A regression-based approach.

[B51-behavsci-15-00181] Hazwani N., Dalbir S., Zulkefli M. (2021). Interface design for e-learning: Investigating design characteristics of colour and graphic elements for generation Z. KSII Transactions on Internet and Information Systems.

[B52-behavsci-15-00181] He J. (2018). Xian dai bao zhuang she ji = Modern packaging design.

[B53-behavsci-15-00181] Heiltjes S. (2014). The effects of multisensory packaging design on brand and product perception and evaluation.

[B54-behavsci-15-00181] Henderson P. W., Cote J. A. (1998). Guidelines for Selecting or Modifying Logos. Joural of Marketing.

[B55-behavsci-15-00181] Henderson P. W., Cote J. A., Leong S. M., Schmitt B. (2003). Building strong brands in Asia: Selecting the visual components of image to maximize brand strength. International Journal of Research in Marketing.

[B56-behavsci-15-00181] Henseler J., Ringle C. M., Sarstedt M. (2015). A new criterion for assessing discriminant validity in variance-based structural equation modeling. Journal of the Academy of Marketing Science.

[B57-behavsci-15-00181] Hess A. C., Melnyk V. (2016). Pink or blue? The impact of gender cues on brand perceptions. European Journal of Marketing.

[B58-behavsci-15-00181] Housni S., Nechouani I., Machrafi M. (2023). Understanding the factors affecting university students’ intention to purchase terroir products: An S-O-R model approach. 97th International Scientific Conference on Economic and Social Development—“Modern Technologies and Innovative Concepts in the Function of Promoting Cultural Heritage”.

[B59-behavsci-15-00181] Hu L., Bentler P. M. (1999). Cutoff criteria for fit indexes in covariance structure analysis: Conventional criteria versus new alternatives. Structural Equation Modeling.

[B60-behavsci-15-00181] Huang Y., Jiang P., Tanaka T. (2023). A study of chinese characters users’ perceived impressions of fonts with the same glyph writing. Design Research.

[B61-behavsci-15-00181] Hurley R. A., Randall R., O’Hara L., Tonkin C., Rice J. C. (2017). Color harmonies in packaging. Color Research & Application.

[B62-behavsci-15-00181] Hwang J., Choe J. Y. (Jacey), Kim H. M., Kim J. J. (2021). Human baristas and robot baristas: How does brand experience affect brand satisfaction, brand attitude, brand attachment, and brand loyalty?. International Journal of Hospitality Management.

[B63-behavsci-15-00181] iiMedia Research (2021). Report on the status quo and consumption trends of China’s teabag industry in the first half of 2021.

[B64-behavsci-15-00181] Ipsos (2020). Tea flavor preferences and consumption patterns of post-90s new consumers.

[B65-behavsci-15-00181] Jager J., Putnick D. L., Bornstein M. H. (2017). II. More than just convenient: The scientific merits of homogeneous convenience samples. Monographs of the Society for Research in Child Development.

[B66-behavsci-15-00181] Jeon H. M., Yoo S. R. (2021). The relationship between brand experience and consumer-based brand equity in grocerants. Service Business.

[B67-behavsci-15-00181] Kan Y. (2012). Design and application of typeface.

[B68-behavsci-15-00181] Kang L. (2014). Shijue chuanda sheji de zaoxing yaosu yu butong lingyu.

[B69-behavsci-15-00181] Kauppinen-Räisänen H. (2014). Strategic use of colour in brand packaging: Strategic use of colour in brand packaging. Packaging Technology and Science.

[B70-behavsci-15-00181] Kelloway E. K. (2015). Using Mplus for structural equation modeling: A researcher’s guide.

[B71-behavsci-15-00181] Khan I., Rahman Z. (2015). A review and future directions of brand experience research. International Strategic Management Review.

[B72-behavsci-15-00181] Khowjoy K., Petmee P., Phakamach V., Sriplang N., Kaewsrem S., Chayomchai A. (2023). Factors influencing brand loyalty: The mediating effect of brand satisfaction and trust. Polish Journal of Management Studies.

[B73-behavsci-15-00181] Kiygi Calli M., Kilic S. (2020). Ürün tercihlerini etkileyen ambalaj tasarim faktörlerinin kismi yarar konjoint analizi ile belirlenmesi: Organik sabun ürünü üzerine bir çalişma. Business and Economics Research Journal.

[B74-behavsci-15-00181] Klimchuk M. R., Krasovec S. A. (2021). Bao zhuang she ji: Cheng gong pin pai de su zao li: Cong gai nian gou si dao huo jia zhan shi=Packaging design: Successful product branding from concept to shelf.

[B75-behavsci-15-00181] Kline R. B. (2004). Beyond significance testing: Reforming data analysis methods in behavioral research.

[B76-behavsci-15-00181] Kline R. B., Little T. D. (2023). Principles and practice of structural equation modeling.

[B77-behavsci-15-00181] Kovačević D., Mešić E., Užarević J., Brozović M. (2022). The influence of packaging visual design on consumer food product choices. Journal of Print and Media Technology Research.

[B78-behavsci-15-00181] Köseoğlu D., Tuncer İ. (2023). The importance of store image in retail food markets: An analysis within the framework of the S-O-R paradigm. Eskişehir Osmangazi Üniversitesi İktisadi ve İdari Bilimler Dergisi.

[B79-behavsci-15-00181] Krejcie R. V., Morgan D. W. (1970). Determining sample size for research activities. Educational and Psychological Measurement.

[B80-behavsci-15-00181] Krishna A., Cian L., Aydınoğlu N. Z. (2017). Sensory aspects of package design. Journal of Retailing.

[B81-behavsci-15-00181] Kumar Mishra M., Kesharwani A., Das D. (2016). The relationship between risk aversion, brand trust, brand affect and loyalty: Evidence from the FMCG industry. Journal of Indian Business Research.

[B82-behavsci-15-00181] Labrecque L. I., Milne G. R. (2012). Exciting red and competent blue: The importance of color in marketing. Journal of the Academy of Marketing Science.

[B83-behavsci-15-00181] Lacoste-Badie S., Gagnan A. B., Droulers O. (2020). Front of pack symmetry influences visual attention. Journal of Retailing and Consumer Services.

[B84-behavsci-15-00181] Li H., Hu J., Sun X. (2023). Comprehensive evaluation of gene sequence encoding methods in deep learning.

[B85-behavsci-15-00181] Liu S. Q., Choi S., Mattila A. S. (2019). Love is in the menu: Leveraging healthy restaurant brands with handwritten typeface. Journal of Business Research.

[B86-behavsci-15-00181] Lupton E., Phillips J. (2009). Graphic design: The new basics.

[B87-behavsci-15-00181] MacGregor L. J., Gilbert R. A., Balewski Z., Mitchell D. J., Erzinçlio S. W., Fedorenko E., Davis M. H. (2022). Causal contributions of the domain-general (multiple demand) and the language-selective brain networks to perceptual and semantic challenges in speech comprehension. Neurobiology of Language.

[B88-behavsci-15-00181] MacKenzie S. B., Podsakoff P. M. (2012). Common method bias in marketing: Causes, mechanisms, and procedural remedies. Journal of Retailing.

[B89-behavsci-15-00181] Maleki S., Amiri Aghdaie S. F., Shahin A., Ansari A. (2020). Investigating the relationship among the Kansei-based design of chocolate packaging, consumer perception, and willingness to buy. Journal of Marketing Communications.

[B90-behavsci-15-00181] Malhotra N. K., Schaller T. K., Patil A. (2017). Common method variance in advertising research: When to be concerned and how to control for it. Journal of Advertising.

[B91-behavsci-15-00181] Martinez L. M., Rando B., Agante L., Abreu A. M. (2021). True colors: Consumers’ packaging choices depend on the color of retail environment. Journal of Retailing and Consumer Services.

[B92-behavsci-15-00181] Montgomery D. C. (2017). Design and analysis of experiments.

[B93-behavsci-15-00181] Moreira A. C., Fortes N., Santiago R. (2017). Influence of sensory stimuli on brand experience, brand equity and purchase intention. Journal of Business Economics and Management.

[B94-behavsci-15-00181] Murchie K. J., Diomede D. (2020). Fundamentals of graphic design—Essential tools for effective visual science communication. FACETS.

[B95-behavsci-15-00181] Nivedhitha K. S., Manzoor A. K. S. (2020). Gamification inducing creative ideation: A parallel mediation model. Behaviour & Information Technology.

[B96-behavsci-15-00181] Nunnally J. C. (1978). Psychometric theory.

[B97-behavsci-15-00181] Nöth W. (1997). Semiotics of the media: State of the art, projects, and perspectives.

[B98-behavsci-15-00181] Ong C. H., Lee H. W., Ramayah T. (2018). Impact of brand experience on loyalty. Journal of Hospitality Marketing & Management.

[B99-behavsci-15-00181] Orth U. R., Malkewitz K. (2008). Holistic package design and consumer brand impressions. Journal of Marketing.

[B100-behavsci-15-00181] Otterbring T., Shams P., Wästlund E., Gustafsson A. (2013). Left isn’t always right: Placement of pictorial and textual package elements. British Food Journal.

[B101-behavsci-15-00181] Oyibo K., Vassileva J. (2020). the effect of layout and colour temperature on the perception of tourism websites for mobile devices. Multimodal Technologies and Interaction.

[B102-behavsci-15-00181] Ozretić Došen Đ., Brkljačić L. (2018). Key design elements of daily newspapers: Impact on the reader’s perception and visual impression. KOME.

[B103-behavsci-15-00181] Pan C., Lei Y., Wu J., Wang Y. (2021). The influence of green packaging on consumers’ green purchase intention in the context of online-to-offline commerce. Journal of Systems and Information Technology.

[B104-behavsci-15-00181] Pan Z., Pan H., Zhang J. (2024). The application of graphic language personalized emotion in graphic design. Heliyon.

[B105-behavsci-15-00181] Pereira C. T. M., De Medeiros A. C., Ventura M. B., Pereira D. M., Bolini H. M. A. (2022). Do the colors of the label and the sweetening agent information influence the sensory expectations consumer? A case study with skyr-type yogurt. Foods.

[B106-behavsci-15-00181] Pleyers G. (2024). Visual complexity in product design: How does the degree of elaborateness of the front-pack image impact consumers’ responses?. Journal of Consumer Behaviour.

[B107-behavsci-15-00181] Podsakoff P. M., Podsakoff N. P., Williams L. J., Huang C., Yang J. (2024). Common method bias: It’s bad, it’s complex, it’s widespread, and it’s not easy to fix. The Annual Review of Organizational Psychology and Organizational Behavior.

[B108-behavsci-15-00181] Poulin R. (2011). The language of graphic design: An illustrated handbook for understanding fundamental design principles.

[B109-behavsci-15-00181] Qi Y., Yan Y. (2020). Influence of multi-channel integration service quality on purchase intention of customers: Dual mediating effect of brand experience and brand trust. Revista Argentina de Clinica Psicologica.

[B110-behavsci-15-00181] Qiao F., Griffin W. G. (2022). Brand imitation strategy, package design and consumer response: What does it take to make a difference?. Journal of Product & Brand Management.

[B111-behavsci-15-00181] Rather R. A., Rasul T., Khan H., Khan I. (2024). Unveiling the dynamics between consumer brand engagement, experience, and relationship quality towards luxury hotel brands: Moderating investigation of brand reputation. International Journal of Hospitality Management.

[B112-behavsci-15-00181] Rehman A. U., Elahi Y. A. (2024). How semiotic product packaging, brand image, perceived brand quality influence brand loyalty and purchase intention: A stimulus-organism-response perspective. Asia Pacific Journal of Marketing and Logistics.

[B113-behavsci-15-00181] Reynolds N. L., Simintiras A. C., Diamantopoulos A. (2003). Theoretical justification of sampling choices in international marketing research: Key issues and guidelines for researchers. Journal of International Business Studies.

[B114-behavsci-15-00181] Robins D., Holmes J., Stansbury M. (2010). Consumer health information on the Web: The relationship of visual design and perceptions of credibility. Journal of the American Society for Information Science and Technology.

[B115-behavsci-15-00181] Rundh B. (2009). Packaging design: Creating competitive advantage with product packaging. British Food Journal.

[B116-behavsci-15-00181] Schmitt B. (1999). Experiential marketing. Journal of Marketing Management.

[B117-behavsci-15-00181] Schuch A. F., Silva A. C. D., Kalschne D. L., Silva-Buzanello R. A. D., Corso M. P., Canan C. (2019). Chicken nuggets packaging attributes impact on consumer purchase intention. Food Science and Technology.

[B118-behavsci-15-00181] Septianto F., Paramita W. (2021). Cute brand logo enhances favorable brand attitude: The moderating role of hope. Journal of Retailing and Consumer Services.

[B119-behavsci-15-00181] Setiowati R., Liem Y. (2018). Impact of packaging design on perceived quality, perceived value, brand preference, and repurchase intention of candy products in Jakarta. Pertanika Journal of Social Sciences & Humanities.

[B120-behavsci-15-00181] Shukla M., Misra R., Singh D. (2022). Exploring relationship among semiotic product packaging, brand experience dimensions, brand trust and purchase intentions in an Asian emerging market. Asia Pacific Journal of Marketing and Logistics.

[B121-behavsci-15-00181] Silayoi P., Speece M. (2004). Packaging and purchase decisions: An exploratory study on the impact of involvement level and time pressure. British Food Journal.

[B122-behavsci-15-00181] Silayoi P., Speece M. (2007). The importance of packaging attributes: A conjoint analysis approach. European Journal of Marketing.

[B123-behavsci-15-00181] Silva J. H. O., Mendes G. H. S., Cauchick-Miguel P. A., Amorim M., Nóvoa H., Drăgoicea M., Kühl N. (2020). Customer experience literature analysis based on bibliometry. Exploring service science.

[B124-behavsci-15-00181] Sin S.-C. J. (2011). Neighborhood disparities in access to information resources: Measuring and mapping U.S. public libraries’ funding and service landscapes. Library & Information Science Research.

[B125-behavsci-15-00181] Sook F. Y., Cheng L. T., Kah B. L., Yong H. K. (2020). Product packaging: Impact on customers’ purchase intention. International Journal of Business and Society.

[B126-behavsci-15-00181] Spence C., Van Doorn G. (2022). Visual communication via the design of food and beverage packaging. Cognitive Research: Principles and Implications.

[B127-behavsci-15-00181] Srivastava P., Ramakanth D., Akhila K., Gaikwad K. K. (2022). Package design as a branding tool in the cosmetic industry: Consumers’ perception vs. reality. SN Business & Economics.

[B128-behavsci-15-00181] Stein A., Ramaseshan B. (2016). Towards the identification of customer experience touch point elements. Journal of Retailing and Consumer Services.

[B129-behavsci-15-00181] Su J., Wang S. (2024). Influence of food packaging color and foods type on consumer purchase intention: The mediating role of perceived fluency. Frontiers in Nutrition.

[B130-behavsci-15-00181] Su K. (2015). The new concept of font basics and applications.

[B131-behavsci-15-00181] Suci A., Maryanti S., Hardi H., Sudiar N. (2022). Willingness to pay for traditional ready-to-eat food packaging: Examining the interplay between shape, font and slogan. Asia Pacific Journal of Marketing and Logistics.

[B132-behavsci-15-00181] Sundström S. (2010). Coding in multiple regression analysis: A review of popular coding techniques.

[B133-behavsci-15-00181] Tang Z., Xu X., Wang F., Zhang L., Zhu M. (2024). Effect of interface layout design of a public library website on information-seeking experience for elderly people. Library Hi Tech.

[B134-behavsci-15-00181] Tasci A. D. A., Milman A. (2019). Exploring experiential consumption dimensions in the theme park context. Current Issues in Tourism.

[B135-behavsci-15-00181] Taylor A. B., MacKinnon D. P., Tein J.-Y. (2008). Tests of the three-path mediated effect. Organizational Research Methods.

[B136-behavsci-15-00181] Theben A., Gerards M., Folkvord F. (2020). The effect of packaging color and health claims on product attitude and buying intention. International Journal of Environmental Research and Public Health.

[B137-behavsci-15-00181] Thomas F., Capelli S. (2023). Increasing purchase intention while limiting binge-eating: The role of repeating the same flavor-giving ingredient image on a front of package. Psychology & Marketing.

[B138-behavsci-15-00181] Titah M. A. (2022). The influence of product packaging and service quality on consumer perception in coffee shop (case study on twenties coffee shop). Jurnal EMBA: Jurnal Riset Ekonomi, Manajemen, Bisnis Dan Akuntansi.

[B139-behavsci-15-00181] Van Esch P., Heller J., Northey G. (2019). The effects of inner packaging color on the desirability of food. Journal of Retailing and Consumer Services.

[B140-behavsci-15-00181] Variawa E. (2010). Buying behaviour and decision-making criteria of Base of the Pyramid consumers: The influence of packaging on Fast Moving Consumer Goods customers’ brand experience.

[B141-behavsci-15-00181] Venter de Villiers M., Chinomona R., Chuchu T. (2018). The influence of store environment on brand attitude, brand experience and purchase intention. South African Journal of Business Management.

[B142-behavsci-15-00181] Vladimirova G. (2017). Typography as a determining factor in the visual communication design. KNOWLEDGE—International Journal.

[B143-behavsci-15-00181] Voorhees C. M., Brady M. K., Calantone R., Ramirez E. (2016). Discriminant validity testing in marketing: An analysis, causes for concern, and proposed remedies. Journal of the Academy of Marketing Science.

[B144-behavsci-15-00181] Waheed S., Khan M. M., Ahmad N. (2018). Product packaging and consumer purchase intentions. Market Forces.

[B145-behavsci-15-00181] Wang E. S.-T. (2013). The influence of visual packaging design on perceived food product quality, value, and brand preference. International Journal of Retail & Distribution Management.

[B146-behavsci-15-00181] Wang E. S.-T. (2017). Different effects of utilitarian and hedonic benefits of retail food packaging on perceived product quality and purchase intention. Journal of Food Products Marketing.

[B147-behavsci-15-00181] Wang F., Wang H., Cho J. H. (2022). Consumer preference for yogurt packaging design using conjoint analysis. Sustainability.

[B148-behavsci-15-00181] Wang J., Wang X. (2020). Structural equation modeling: Applications using Mplus.

[B149-behavsci-15-00181] Wee S.-C., Choong W.-W. (2019). Gamification: Predicting the effectiveness of variety game design elements to intrinsically motivate users’ energy conservation behaviour. Journal of Environmental Management.

[B150-behavsci-15-00181] Wei S.-T., Ou L.-C., Luo M. R., Hutchings J. B. (2014). Package design: Colour harmony and consumer expectations. International Journal of Design.

[B151-behavsci-15-00181] Wen Z. (2024). The application of communication art in tea packaging design under the modern aesthetic perspective. Applied Mathematics and Nonlinear Sciences.

[B152-behavsci-15-00181] Wheeler A. (2021). Designing brand identity.

[B153-behavsci-15-00181] Williamson R. (2019). How does it all unfold? The role of format layout in the user experience of medical instructions for use. Proceedings of the International Symposium on Human Factors and Ergonomics in Health Care.

[B154-behavsci-15-00181] Wong W. (1993). Principles of form and design.

[B155-behavsci-15-00181] Wulansari A. S. (2019). Food product packaging design as marketing tools in purchase decision. Journal of Management and Leadership.

[B156-behavsci-15-00181] Yang Y., Chen Y. (2010). An exploration of customer experience in resort hotels and its empirical enlightenment. Tourism Tribune.

[B157-behavsci-15-00181] Yasri Y., Susanto P., Hoque M. E., Gusti M. A. (2020). Price perception and price appearance on repurchase intention of Gen Y: Do brand experience and brand preference mediate?. Heliyon.

[B158-behavsci-15-00181] Yu Y., Sun Z., Feng C., Xiao X., Hou Y. (2023). The effect of vice–virtue bundles on consumers’ purchase intentions for vice packaged foods: Evidence from randomized experiments. Foods.

[B159-behavsci-15-00181] Yung X. Y. (2023). The positive role of packaging in consumer behavior. Advances in Economics, Management and Political Sciences.

[B160-behavsci-15-00181] Zafar M. Z., Shi X., Yang H., Abbas J., Chen J. (2022). The impact of interpretive packaged food labels on consumer purchase intention: The comparative analysis of efficacy and inefficiency of food labels. International Journal of Environmental Research and Public Health.

[B161-behavsci-15-00181] Zhang F. (2009). History of foreign modern design.

[B162-behavsci-15-00181] Zhao A., Wei W., Choi A. Y., Martins N., Brandão D. (2024). Empirical analysis of packaging design and consumer preferences in the New York Kimchi Market. Advances in design and digital communication IV.

